# *Lacticaseibacillus casei* AMBR2 modulates the epithelial barrier function and immune response in a donor-derived nasal microbiota manner

**DOI:** 10.1038/s41598-020-73857-9

**Published:** 2020-10-09

**Authors:** Charlotte De Rudder, Cristina Garcia-Tímermans, Ilke De Boeck, Sarah Lebeer, Tom Van de Wiele, Marta Calatayud Arroyo

**Affiliations:** 1 Center for Microbial Ecology and Technology, Faculty of Bioscience Engineering, Coupure Links 653, Ghent University, 9000 Ghent, Belgium; 2 Research Group of Environmental Ecology and Applied Microbiology, Department of Bioscience Engineering, University of Antwerp, Antwerp, Belgium; 3 Group of Lactic Bacteria and Probiotics, Department of Biotechnology, Institute of Agrochemistry and Food Technology (IATA), Spanish Research Council (CSIC), Valencia, Spain

**Keywords:** Microbiome, Experimental models of disease, Mucosal immunology

## Abstract

Live biotherapeutic products (LBP) are emerging as alternative treatment strategies for chronic rhinosinusitis. The selection of interesting candidate LBPs often involves model systems that do not include the polymicrobial background (i.e. the host microbiota) in which they will be introduced. Here, we performed a screening in a simplified model system of upper respiratory epithelium to assess the effect of nasal microbiota composition on the ability to attach and grow of a potential LBP, *Lacticaseibacillus casei* AMBR2, in this polymicrobial background. After selecting the most permissive and least permissive donor, *L. casei* AMBR2 colonisation in their respective polymicrobial backgrounds was assessed in more physiologically relevant model systems. We examined cytotoxicity, epithelial barrier function, and cytokine secretion, as well as bacterial cell density and phenotypic diversity in differentiated airway epithelium based models, with or without macrophage-like cells. *L. casei* AMBR2 could colonize in the presence of both selected donor microbiota and increased epithelial barrier resistance in presence of donor-derived nasal bacteria, as well as anti-inflammatory cytokine secretion in the presence of macrophage-like cells. This study highlights the potential of *L. casei* AMBR2 as LBP and the necessity to employ physiologically relevant model systems to investigate host–microbe interaction in LBP research.

## Introduction

The human upper respiratory tract (URT), and more specifically the sinonasal cavities are in constant contact with the outside environment and its physical, chemical and infectious agents. The epithelium is however not alone in facing these potentially harmful external compounds; the human sinonasal cavities are colonised by a diverse microbial community that, in health, aids in maturation of the epithelium, immune priming, and can act as a colonisation barrier towards incoming infectious agents^1–7^. Loss of the balance in microbial composition or functionality (e.g. through increased variability or fragmentation of microbial networks, loss of alpha diversity, increased pathogen abundance, intramucosal invasion), also referred to as dysbiosis, is associated with negative health status^8–11^. A disrupted airway microbiota could render the epithelium more susceptible to pathogen infection and overgrowth, resulting in inflammatory responses^12,13^.

Microbial dysbiosis is a potential etiopathologic factor contributing to chronic rhinosinusitis (CRS). Chronic rhinosinusitis is a disease that affects between 3 and 12% of the adult population in Europe^14–16^. It is defined as chronic inflammation (> 12 weeks) of the nasal and paranasal sinus mucosa, characterised by two or more symptoms, one of which is either nasal obstruction, congestion or nasal discharge and is further identified by facial pain, pressure and/or reduction or loss of smell^17^. Despite the high prevalence of CRS, there is no consensus about the aetiology of the disease (reviewed in^8,18^), and it is currently regarded as an umbrella term for several sub- and endotypes of the disease^19,20^. Historically, CRS was seen as an infection of the sinonasal epithelium and patients are therefore still often treated with antibiotics, despite the lack of high-quality scientific evidence supporting the use of systemic or topical antibiotics in CRS^17,21,22^ and the fact that antibiotic treatment should only be considered in specific cases of CRS^20^. This inappropriate use of antibiotics may increase the spread of antibiotic resistance genes and can result in adverse side effects in patients, such as gastrointestinal discomfort and skin rashes (reviewed in^21^). In recent years, various studies on the association between the sinonasal microbiome and CRS etiopathology and persistence have revealed new insights in the role of bacteria in CRS^11,23–29^. A number of pathogenic bacterial species has been associated with CRS exacerbations and persistence, notably *Staphylococcus aureus*, *Pseudomonas aeruginosa*, *Streptococcus pneumoniae*, and *Haemophilus influenzae*^29–32^. It is not straightforward to summarise the findings of the different studies, however, most studies point towards an overall dysfunctional interaction between the sinonasal epithelium and its resident microbiota. Restoring this dysfunctional interaction is thus a major objective to restore host health^27,33,34^. The use of probiotics or live biotherapeutic products (LBPs) has been proposed as a possible strategy to restore the balanced interaction between host and microbiota in the sinonasal cavities by reinforcing beneficial bacteria of the microbiota, strengthen the epithelial barrier, and reduce inflammation^34^.

LBPs are biological products that: (1) contain live organisms, such as bacteria; (2) are applicable to the prevention, treatment, or cure of a disease or condition of human beings; and (3) are not vaccines^35^. They form a special class of probiotics, which are defined as live microorganisms that, when applied in adequate amounts, confer a health benefit to the host. They are an emerging alternative strategy to treat upper respiratory tract diseases^33,34,36–40^. Their mechanisms of action can be divided in the exclusion or inhibition of pathogens, improvement of epithelial barrier function, and modulation of local or systemic host immune responses^34^. De Boeck et al.^41^ have demonstrated that certain lactobacilli are reduced in the nasopharynx of CRS patients compared to healthy controls. One particular *Lacticaseibacillus casei* strain, the species formerly known as *Lactobacillus casei*^42^, strain AMBR2, was isolated from the URT of a healthy volunteer. This strain was shown to express fimbriae enabling strong adherence to URT epithelium and inhibit the growth and virulence of several URT pathogens. These adaptive traits that enable survival and colonisation in the human URT could potentially increase the efficacy of *L. casei* AMBR2 as live biotherapeutic product. Indeed, an explorative first in-human study showed that the strain could persist in the nose of healthy volunteers up to 2 weeks after the last administration, thus longer than the nasal clearance time of 15 min^41^, but a detailed study on the colonisation dynamics of this strain has not yet been performed.

The screening and efficacy testing of potential LBPs for the respiratory tract generally starts in cell culture models as pure strains^41^ and^43^, and/or in simple competition experiments with pathogenic species^40,41,44,45^. However, one of the first biological barriers for the successful engraftment of an LBP is formed by the resident microbiota^46^. When nasally applied as a (prophylactic) treatment, these strains will be introduced in an environment colonised by an existing microbial community, with interindividual differences in composition^11,47–50^, microbial load^23,51^, and, especially in case of a pathogen-dominated dysbiosis, potentially permissiveness to engraftment^52–54^. In the gut, the mucosal colonisation permissiveness of probiotics has been shown to be highly person-specific, with both the microbiota and the immune responses determining successful colonisation^55,56^. In order to better predict the efficacy and responders of LBPs, it is important to assess the potency of novel LBPs in a microbial community that is more representative for the one that will be encountered upon administration, including interindividual variability. Few in vitro host–microbe interaction models of the sinonasal cavities include natural nasal microbiota^57,58^.

We have previously developed dual and triple co-culture in vitro model systems to mimic and study host–microbe interactions in the human URT microenvironment for 72 h using models containing respectively differentiated Calu-3 respiratory epithelial cells apically inoculated with donor-derived microbiota (dual co-culture), and the former combination with the addition of THP-1 derived macrophage-like cells (triple co-culture)^58^. The differentiation of a Calu-3 cell layer at the air–liquid interface (ALI) results in an epithelial layer with functioning epithelial barrier, ciliated cells and mucus-producing goblet cells^59–62^. The inclusion of THP-1 derived macrophage-like cells allows to study crosstalk between epithelial cells, bacteria, and macrophages^63^.

**Figure 1:**
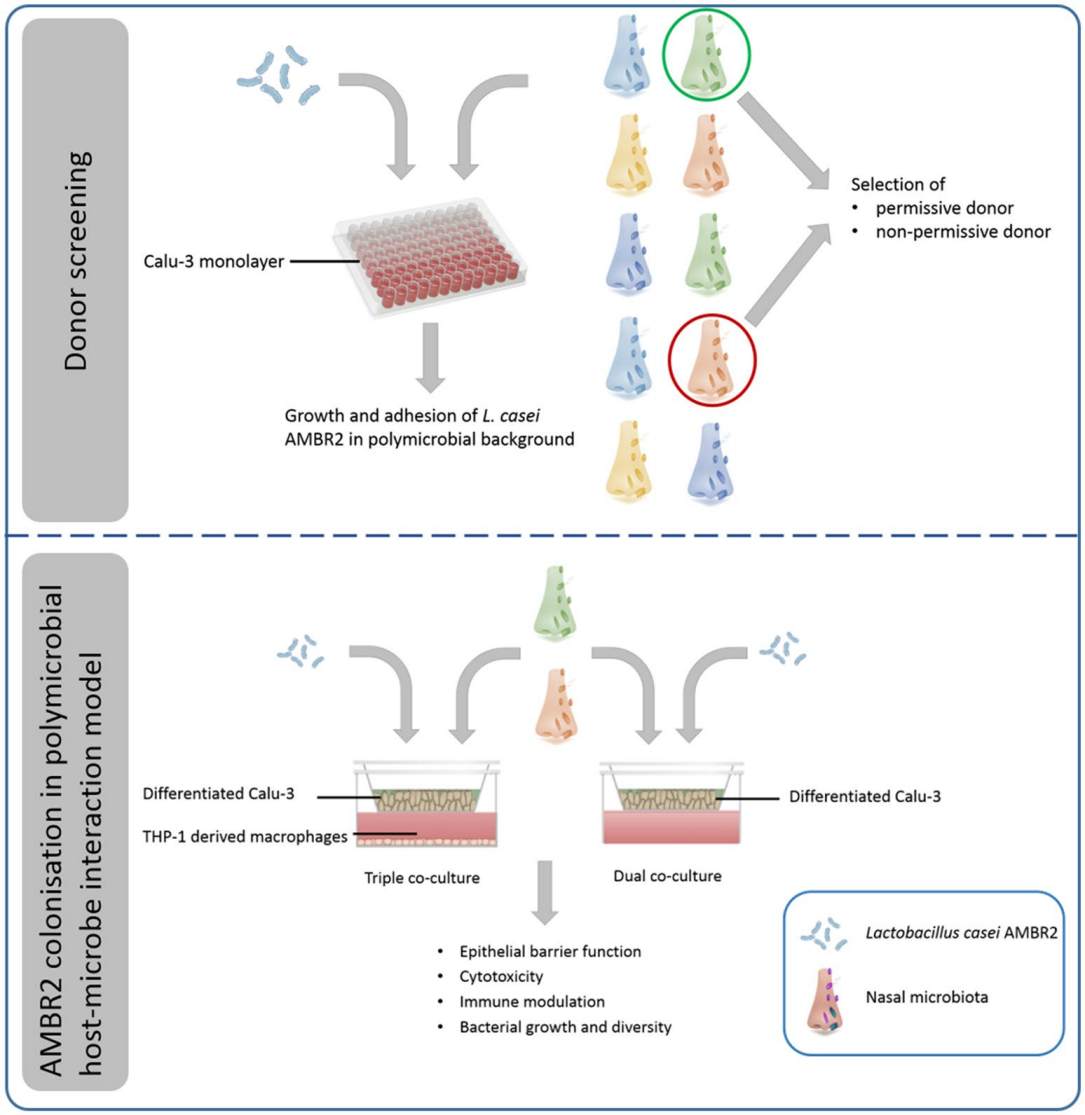
Schematic representation of screening for donor permissiveness to *L. casei* AMBR2 colonisation and assessment of *L. casei* AMBR2 probiotic potency in complex host–microbe interaction models.

In this study, we examined the colonisation potential and host–microbe interaction of *L. casei* AMBR2 in donor-derived polymicrobial background, as presented in Fig. 1. In an initial screening, we assessed growth and attachment of *L. casei* AMBR2 on submerged respiratory epithelial monolayers in the presence of nasal microbial communities obtained from 10 different healthy donors. Two donors were selected from this group based on the ability of AMBR2 to grow in their nasal communities. A sample of their nasal microbiota was used to inoculate dual and triple co-culture models, which were then treated with *L. casei* AMBR2. We evaluated the colonisation behaviour of *L. casei* AMBR2 and the host responses in these complex model systems, and the manner in which the inclusion of immune cells impacted bacterial and epithelial behaviour.

## Results

### Donor screening

#### *L. casei* AMBR2 survival and growth in polymicrobial and host cell background

**Figure 2:**
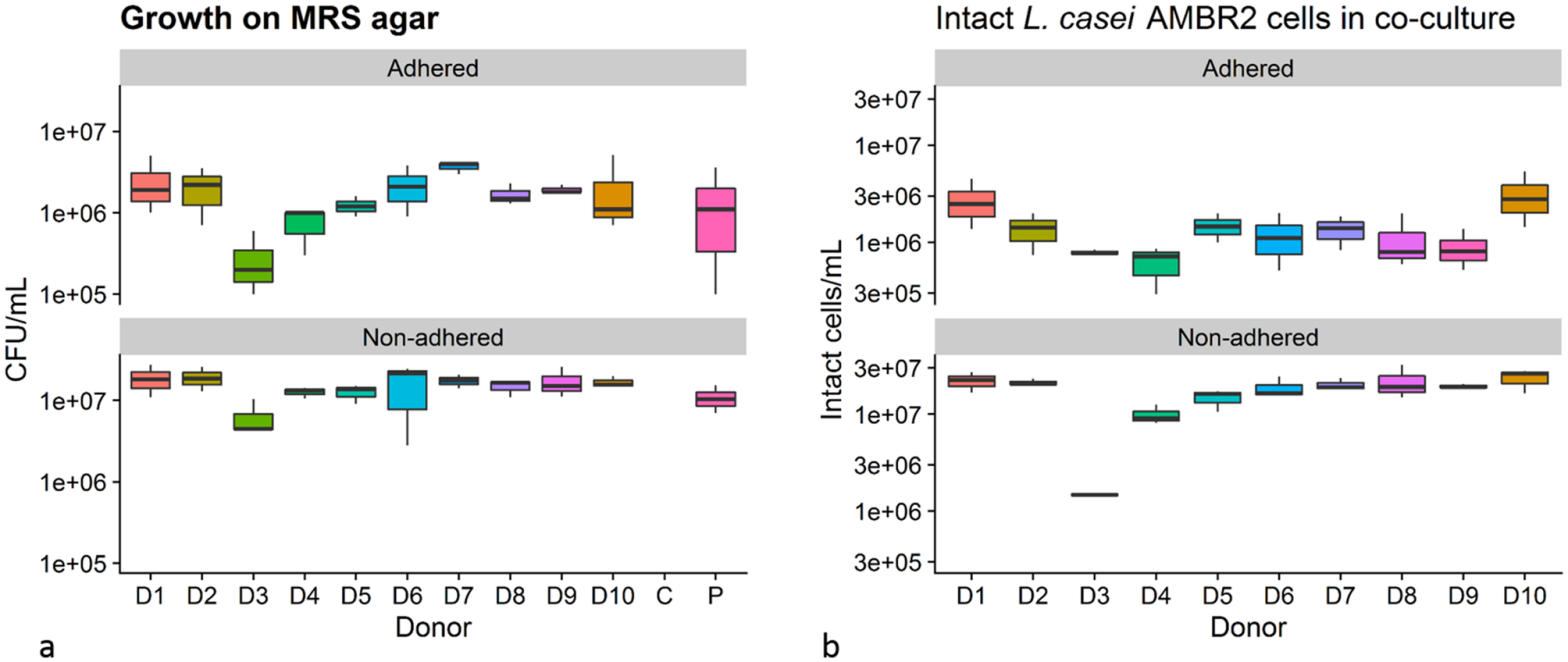
(**a**) Growth of lactic acid bacteria 24 h after inoculation of 100 µL of 10^8^ intact *L. casei* AMBR2 cells on respiratory cell monolayers in polymicrobial backgrounds as calculated from MRS plate colony counts. (**b**) Intact *L. casei* AMBR2 cell density as calculated using flow cytometry data. Donors are indicated with numbers 1–10, C = sterile control, P = *L. casei* AMBR2 only. ‘Adhered’ refers to bacteria that were adhered to Calu-3 cell layers (n = 3 for each test group), ‘Non-adhered’ refers to bacteria retrieved from the apical medium (n = 3 for each test group).

24 h after inoculation, we separately sampled bacteria that were adhered or non-adhered to Calu-3 respiratory epithelial cell monolayers, as adherence to the epithelial cell layer is an important trait to avoid rapid mucociliary clearance of LBP in the URT. Adhered and non-adhered bacteria were plated on MRS agar to determine the amount of cultivable lactic acid bacteria in the samples. No colonies were found on plates with samples from nasal microbiota without probiotic, or on the sterile control group. Growth occurred in all samples from wells inoculated with *L. casei* AMBR2, as displayed in Fig. 2a. We found no significant differences in growth in polymicrobial backgrounds from the different donors. The number of adhered lactic acid bacteria ranged from 10^4^ CFU mL^−1^ to 5.10 × 10^6^ CFU mL^−1^ with an average of 1.89 × 10^6^ CFU mL^−1^ ± 1.39 × 10^6^ CFU mL^−1^ (standard deviation), 11.06% of the total (adhered + non-adhered) colony forming community on MRS agar. Non-adhered lactic acid bacteria ranged from 2.80 × 10^6^ CFU mL^−1^ to 2.73 × 10^7^ CFU mL^−1^ with an average of 1.52 × 10^7^ CFU mL^−1^ ± 6.20 × 10^6^ CFU mL^−1^, 88.94% of the total colony forming community. In samples with *L. casei* AMBR2 without polymicrobial background, lactic acid bacteria grew to an average of 1.60 × 10^6^ CFU mL^−1^ ± 1.80 × 10^6^ CFU mL^−1^ adhered cells (12.5% of the total colony forming community) and 1.12 × 10^7^ CFU mL^−1^ ± 5.94 × 10^7^ CFU mL^−1^ non-adhered cells (87.5% of the total colony forming community) in the medium. Donor 3 and donor 7 were selected for further testing based on having respectively the lowest and the highest average attached colony counts on MRS agar.

**Figure 3:**
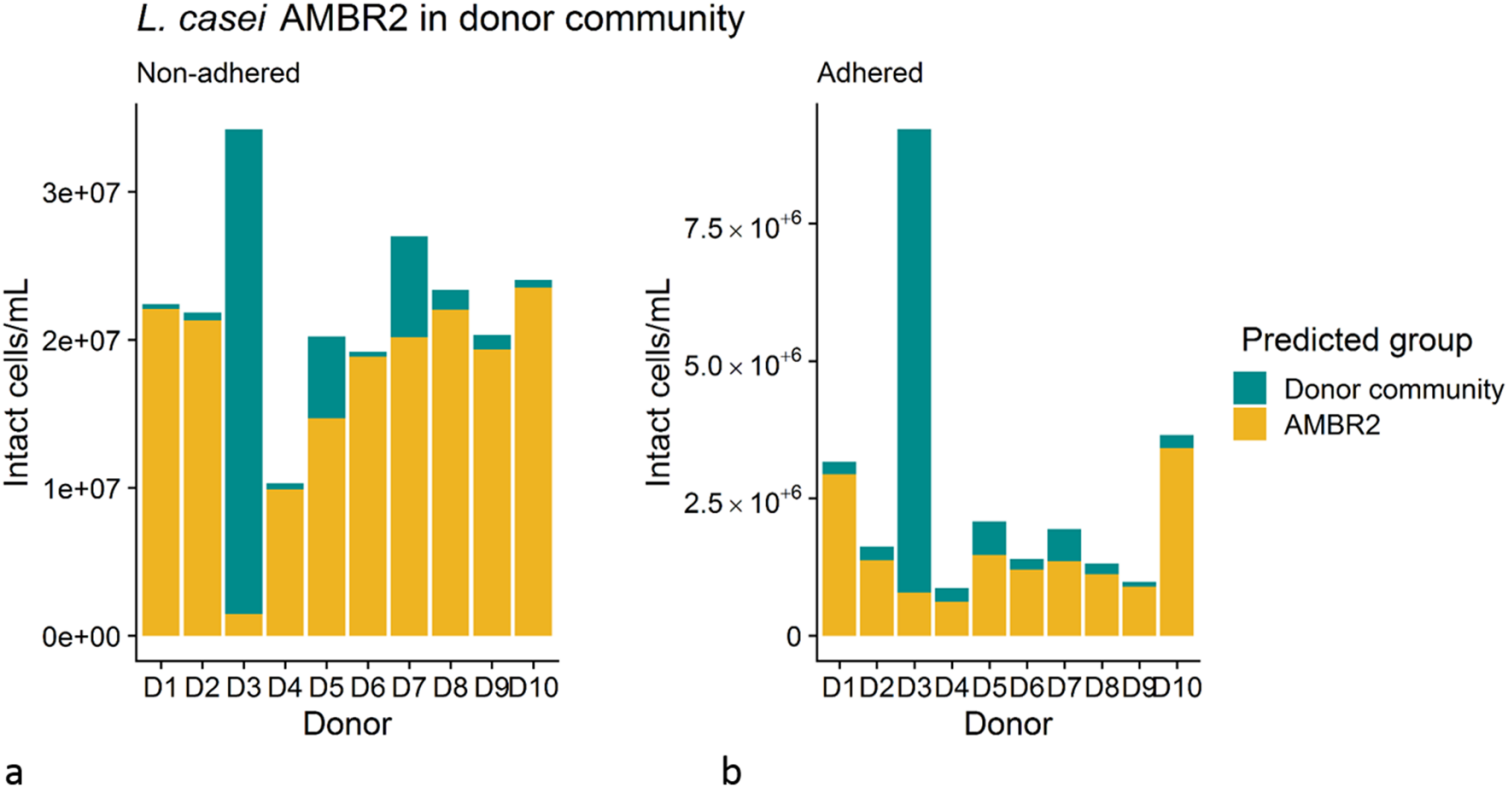
Non-adhered (**a**) and adhered (**b**) *L. casei* AMBR2 and donor community intact cell concentration, as predicted using a Random Forest classifier on flow cytometry data. D1–D10 indicates donors 1–10. Bars represent the average of three samples for each donor.

We used Random Forest classifiers to further estimate the number of intact *L. casei* AMBR2 cells in the adhered and non-adhered polymicrobial donor-derived communities (Figs. 2b, 3), which corresponded to the lactic acid bacteria count we observed using selective plating. This indicated that *L. casei* AMBR2 could survive or grow in all donor backgrounds, except for donor 3, where a decrease in non-adhered intact cell counts compared to the inoculum was observed. Incubation with donor 3 derived microbiota resulted in significantly less estimated non-adhered intact AMBR2 cells (K–W, *p* = 0.03; Wilcoxon tests, *p* = 0.05) than with other donor backgrounds. This trend was observed, yet non-significant, in non-adhered microbial communities as well.

#### Bacterial growth and phenotypic diversity

**Figure 4:**
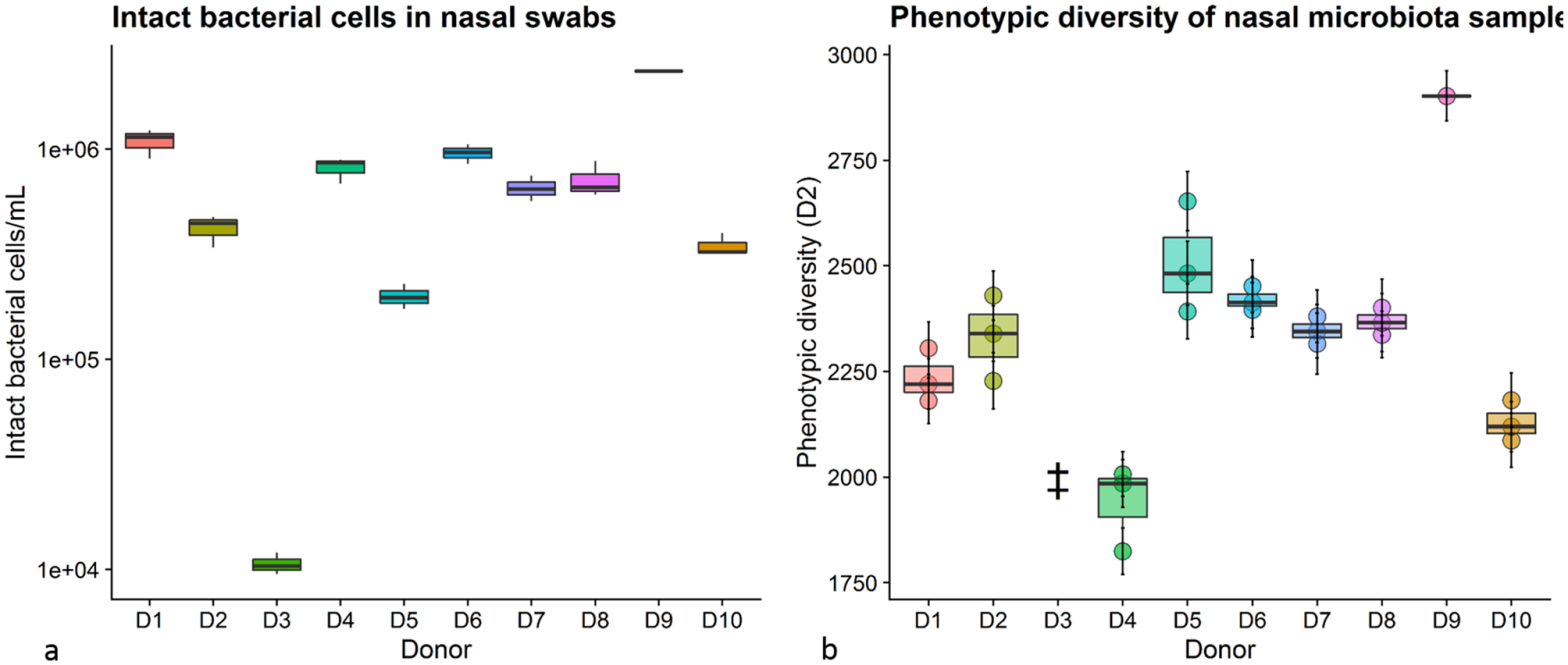
(**a**) Intact bacterial cell counts in nasal swab samples, as measured by flow cytometry after intact/damaged staining. Boxplots are based on three technical replicates, except for donor 9 (n = 1). (**b**) Phenotypic diversity in nasal swabs as calculated based on flow cytometry data. Boxplots are based on three technical replicates, except for donor 9 (n = 1). Double dagger indicates donor 3 for which bacterial density was too low to calculate phenotypic diversity.

To explore the microbial community factors that could account for differences in facilitating the growth or survival of *L. casei* AMBR2, we evaluated bacterial cell density and phenotypic diversity in the nasal swab communities used to inoculate the epithelial cell monolayers during the donor screening. The bacterial cell density in the swab samples obtained from the donor’s nasal cavities, is displayed in Fig. 4a. While average intact cell density in the nasal samples was 2.69 × 10^5^ intact cells mL^−1^ ± 1.98 × 10^5^ cells mL^−1^, donor 3 displayed a bacterial density of 3.98 × 10^3^ intact cells mL^−1^ ± 1.19 × 10^3^ cells mL^−1^, which was lower (*p* = 0.05, Wilcoxon test) than all other donors, except for donor 9. Donor derived inocula were brought to the same concentration prior to inoculation to evaluate the effect of community composition rather than cell density. Phenotypic diversity was calculated based on intact bacterial cell flow cytometric data (10^3^ events/sample), and is displayed in Fig. 4b. Intact bacterial cell densities in samples from donor 3 were too low to calculate phenotypic diversity.

**Figure 5:**
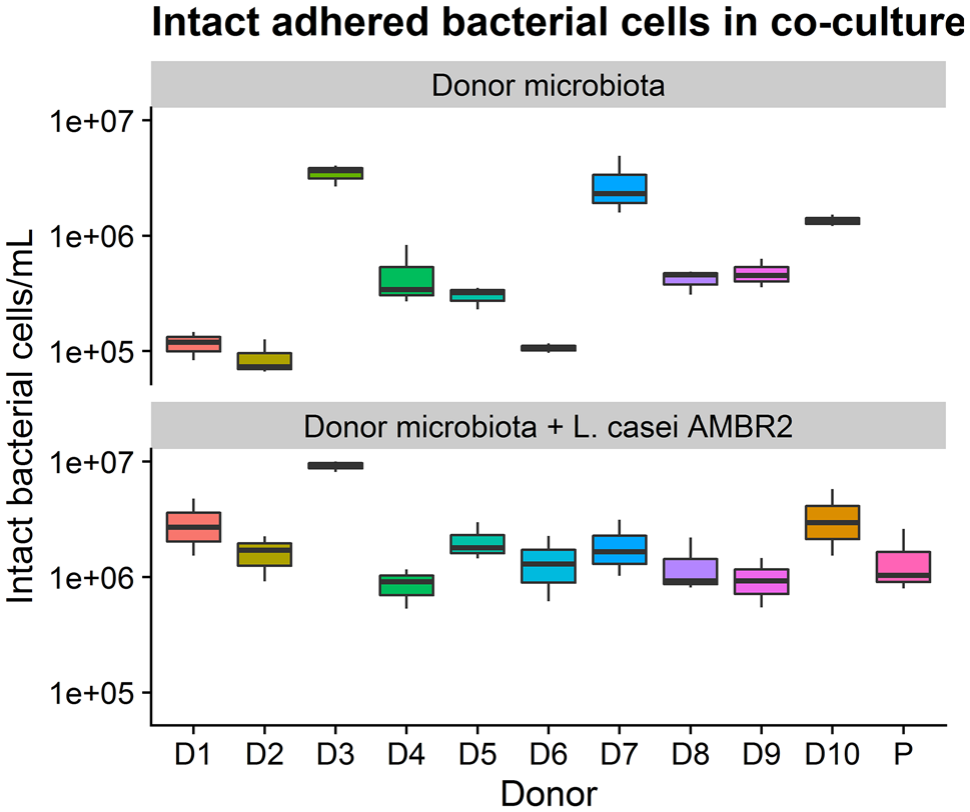
Intact adhered bacterial cells after 24 h of co-culture on a respiratory epithelial cell monolayer, as determined by flow cytometry after intact/damaged staining. D1-D10 indicates donors 1–10, P indicates *L. casei* AMBR2.

The density of intact bacteria adhered to the Calu-3 monolayers after 24 h of co-culture with or without *L. casei* AMBR2 was measured using flow cytometry (Fig. 5). On average, bacterial cell densities with AMBR2 were 2.46 × 10^6^ intact cells mL^−1^ ± 2.54 × 10^6^ cells mL^−1^. Adhered intact cell densities without *L. casei* AMBR2 were on average 9.75 × 10^5^ intact cells mL^−1^ ± 1.29 × 10^6^ intact cells mL^−1^, and showed large variation between donors.

Intact non-adhered bacterial cell densities were measured after 24 h of incubation on Calu-3 monolayers with or without *L. casei* AMBR2. On average, bacterial cell densities with AMBR2 were 6.8 × 10^7^ intact cells mL^−1^ ± 1.6 × 10^8^ intact cells mL^−1^. Further analysis showed that for all donors, except for donor 3, and the probiotic alone, average intact cell density was 2.16 × 10^7^ intact cells mL^−1^ ± 4.68 × 10^6^ intact cells mL^−1^, whereas for donor 3, average intact cell density was 5.36 × 10^8^ intact cells mL^−1^ ± 3.18 × 10^7^ intact cells mL^−1^, more than tenfold higher in comparison with the other donors. Donor 3 with AMBR2 had significantly higher cell densities than other donors with AMBR2, except for donor 1 and 10 (Wilcoxon Rank Sum tests). Overall differences were however not significant (K–W). Without AMBR2, average intact cell counts were 9.79 × 10^7^ intact cells mL^−1^ ± 2.45 × 10^8^ CFU mL^−1^, ranging from 1.26 × 10^5^ intact cells mL^−1^ (donor 1) to 8.31 × 10^8^ intact cells mL^−1^ (donor 3). We observed significant differences between donors in bacterial communities without AMBR2 (K–W, *p* = 0.001506); donor 3 and donor 7 had higher bacterial cell densities than all other donors (Wilcoxon Ranked Sum tests).

**Figure 6:**
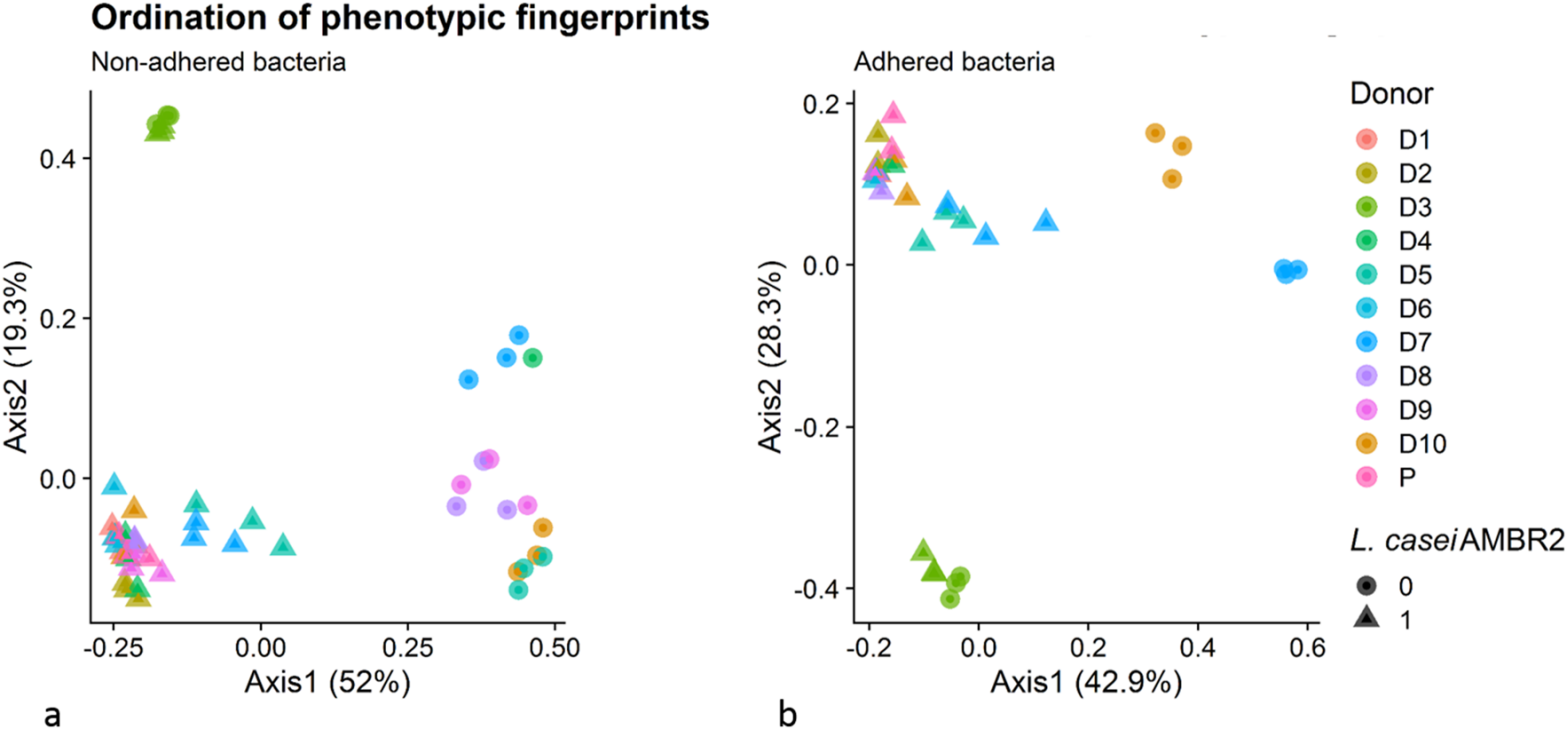
PCoA of phenotypic fingerprints of non-adhered (**a**) and adhered (**b**) bacteria after 24 h incubation on Calu-3 monolayers with (triangle) or without (circle) AMBR2. D1–D10 indicates donors 1–10, P indicates *L. casei* AMBR2. D1–D10 indicates donors 1–10, P indicates *L. casei* AMBR2.

Phenotypic *β*-diversity analysis on adhered and non-adhered microbial communities demonstrated that except for donor 3, microbial communities inoculated with *L. casei* AMBR2 clustered together (PCoA, Fig. 6a,b), in line with the *Lacticaseibacillus*-dominated communities observed after partial 16S rRNA gene sequencing (Supplementary Figure 1). Communities derived from donor 3 with or without *L. casei* AMBR2 clustered separately from all other communities.

#### Phylogenetic microbial community analysis

**Figure 7:**
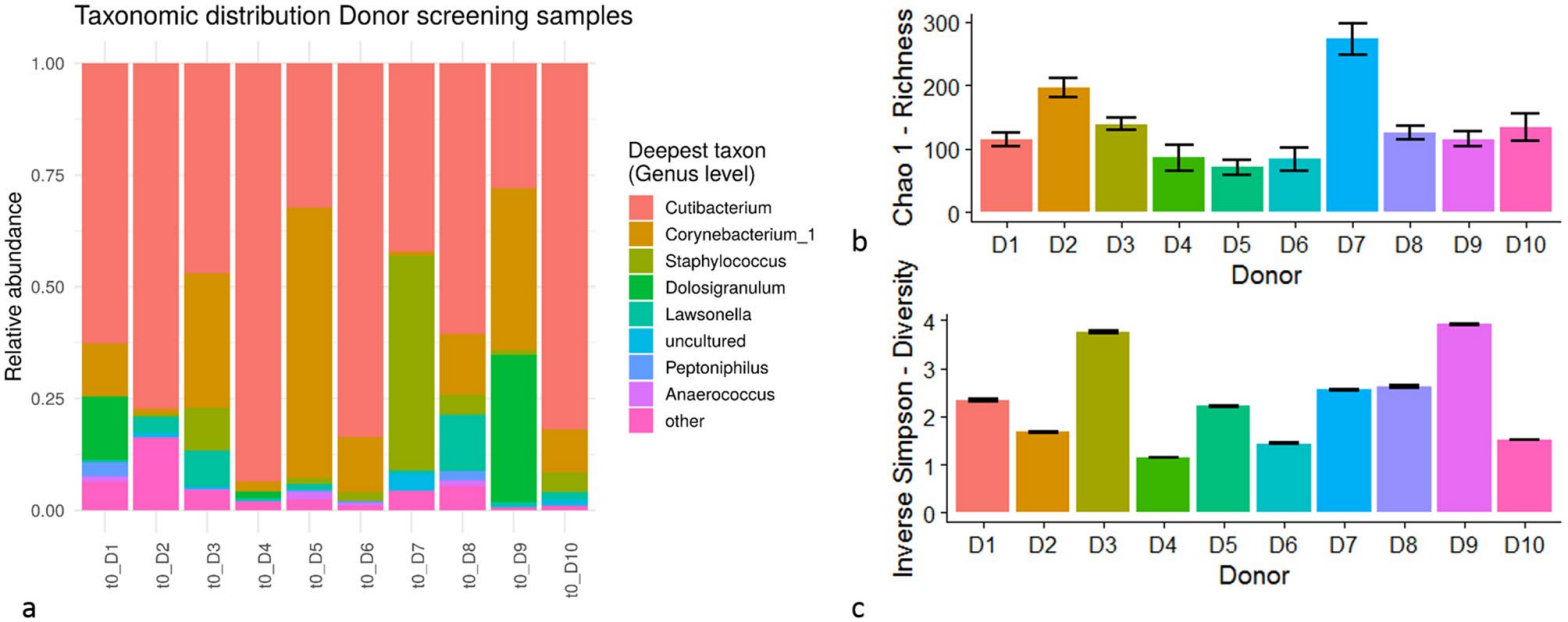
(**a**) Relative abundance on genus level of the eight most abundant genera. (**b**) Chao1 richness of nasal swab bacterial communities. (**c**) Inverse Simpson Diversity of nasal swabs. Error bars indicate standard deviation. D1–D10 indicates donors 1–10, P indicates *L. casei* AMBR2. D1–D10 indicates donors 1–10, P indicates *L. casei* AMBR2.

The relative abundances of the eight most abundant genera present in the nasal bacterial communities of the donors are displayed in Fig. 7a. Nasal swab bacterial community richness was estimated by the Chao1 index (Fig. 7b) and the community alpha-diversity was calculated using the Inverse Simpson index (Fig. 7c). An overview of richness and diversity in the different sample types and treatment groups is given in Table 1. Richness was significantly increased in cultured samples, with and without AMBR2 (Wilcoxon test, *p* < 0.01), whereas diversity was decreased after 24 h of culture (Wilcoxon test, *p* < 0.001). In the adhered bacterial communities, introduction of AMBR2 resulted in an increase in richness (*p* = 0.017, Wilcoxon test), but not diversity. In the non-adhered communities, introduction of AMBR2 did not result in significant differences in richness or diversity.

**Table 1:** Chao1 index for richness and Inverse Simpson index for diversity of the bacterial community, as determined by partial 16S rRNA gene sequencing. AMBR2 indicates *L. casei* AMBR2 presence (1) or absence (0). Chao1 and Inverse Simpson indices are expressed as average ± standard deviation (n = 10 for each treatment).

Time [h]	Sample type	AMBR2	Chao1 richness	Inverse Simpson diversity
0	Swab	0	133.6 ± 60.3	2.3 ± 0.9
24	Adhered	0	303.3 ± 91.1	1.1 ± 0.3
24	Adhered	1	495.0 ± 175.0	1.4 ± 0.4
24	Non-adhered	0	333.8 ± 182.5	1.4 ± 1.0
24	Non-adhered	1	472.3 ± 230.9	1.2 ± 0.2

Taxa from the *Lactobacillus* genus complex, likely *L. casei* AMBR2, were able to attach and survive in all donor communities; after 24 h *Lactobacillus* sp. had a relative abundance of 76.7% ± 34.3% (average ± standard deviation) in the non-adhered communities, and 70.7% ± 33.7% in the adhered communities in which it was introduced (Supplementary Figure 1). The consensus sequence of the most abundant *Lactobacillus* OTU, OTU2, was compared to the expanded Human Oral Microbiome Database (eHOMD^54^), which indicated that it was *Lactobacillus paracasei*, reclassified as *Lacticaseibacillus paracasei*^42^ (100% identity ), or *Lactobacillus casei*, reclassified as *Lacticaseibacillus casei*^42^ (99.8% identity). In 7 out of 10 communities where AMBR2 was not introduced, an overgrowth of *Staphylococcus* sp. occurred (OTU1). This genus (represented by OTU1) was also the second most abundant (after *Lactobacillus* sp.) in 8 out of 10 communities where AMBR2 was introduced. Because *Staphylococcus* taxa can be important pathobionts in CRS^64,65^, we assessed whether there was a correlation between the initial relative abundance of *Staphylococcus* sp. and the relative abundance of *Lactobacillus* sp. after 24 h. Both Spearman (r_S_ = − 0.68, *p* = 0.029, n = 10) and Pearson (r_P_ = − 0.62, *p* = 0.054, n = 10) correlation indicated a negative relationship between the abundances of both genera. Comparison of the consensus sequence of the partial 16S rRNA gene with eHOMD^54^ indicated OTU1 was *Staphylococcus epidermidis* (identity = 100%).

A notable exception was the bacterial community of donor 3, where *Lactobacillus* sp. made up only 1.37% (non-adhered) and 2.06% (adhered) of the relative abundance 24 h after introduction of AMBR2, whereas unclassified *Enterobacteriaceae* (OTU4) accounted for 95.5% (non-adhered) and 94.4% (adhered) of the relative abundance in these communities. At the start of the experiment, donor 3 had 0.53% unclassified *Enterobacteriaceae* in their nasal community. Two other donors, 8 and 10, had respectively 0.04% and 0.29% unclassified *Enterobacteriaceae* (OTU4) in their nasal community. However, after 24 h, their communities were dominated by *Staphylococcus* sp. (without AMBR2) or *Lactobacillus* sp. (with AMBR2). We compared the consensus sequence of the partial 16S rRNA gene with eHOMD^54^, which indicated OTU4 was *Klebsiella aerogenes* (identity = 100%).

### Colonisation of *L. casei* AMBR2 in dual and triple upper respiratory tract host–microbe interaction set-ups with selected donor microbial backgrounds

#### Cytotoxicity

Cytotoxic stress of Calu-3 or Calu-3 and THP-1 cells exposed to bacterial communities was assessed through lactate dehydrogenase (LDH) leakage to the medium in the basal compartment (Supplementary Figure 2). Cytotoxicity remained below 5% for all co-cultures and control groups throughout the experiment. The donor background had no significant effect on cytotoxicity, nor did treatment with *L. casei* AMBR2 alter LDH release. The presence of macrophages resulted in a small yet significant reduction in percentage cytotoxicity (ANOVA followed by Tukey HSD, *p* < 0.0001).

#### Transepithelial electrical resistance

**Figure 8:**
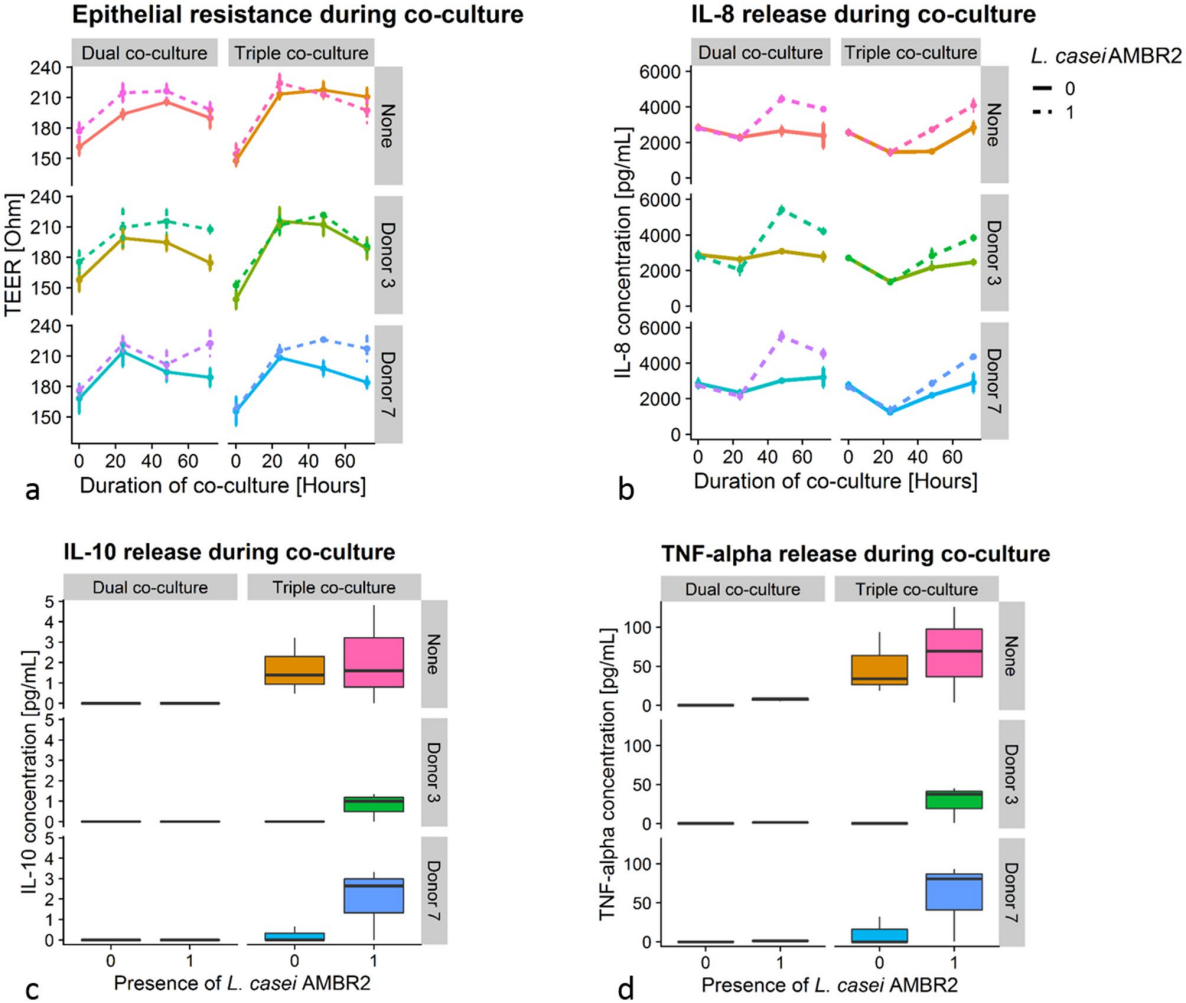
Host responses in dual and triple co-culture systems inoculated with nasal microbiota and/or *L. casei* AMBR2. (**a**) Epithelial electrical resistance in Ohm. (**b**) IL-8 release in basolateral medium as determined by ELISA. (**c**) IL-10 release in basolateral medium, 72 h after inoculation, as determined by ELISA. (**d**) TNF-alpha release in basolateral medium, 72 h after inoculation, as determined by ELISA. “None” indicates the absence of a donor-derived polymicrobial background. 0 and 1 indicate respectively absence and presence of *L. casei* AMBR2.

The epithelial barrier function was measured throughout the experiment as transepithelial electrical resistance (TEER), as displayed in Fig. 8a. At the start of the experiment, the TEER of co-cultures without THP-1 derived macrophages was significantly higher than co-cultures with THP-1 derived macrophages, while after 48 h, the opposite occurred (Wilcoxon Rank Sum test). There were no statistically significant differences in TEER between dual and triple co-cultures 24 h and 72 h after the start of the incubation. 72 h after the start of the experiment, cell layers treated with *L. casei* AMBR2 had a higher epithelial resistance than cell layers without the probiotic (Wilcoxon Rank Sum test). Looking at the different donor backgrounds, we observed that dual and triple co-culture systems inoculated with donor microbiota from donor 7 treated with *L. casei* AMBR2 had higher epithelial resistances than their counterparts without AMBR2 (K–W). For donor 3, this effect of AMBR2 only occurred in dual co-culture (K–W). Without donor background, no such effect of AMBR2 treatment was observed (K–W).

#### Cytokine secretion

We evaluated release of pro- and anti-inflammatory cytokines in response to the nasal microbiota and *L. casei* AMBR2 to investigate the effect of *L. casei* AMBR2 on inflammation or immune surveillance. Incubation with *L. casei* AMBR2 had an impact on the IL-8 release in both model systems and for both donor backgrounds (K–W, *p* = 0.001335, Fig. 8b). In dual co-culture, IL-8 release in AMBR2 treated groups showed to be slightly but significantly lower after 24 h than in non-treated groups (KW, *p* = 0.00919). From 48 h after inoculation on, AMBR2 treated cell cultures released significantly more IL-8 than non-treated cell cultures (K–W, *p* = 0.00035 for both model systems at 48 h and 72 h). While this increase in IL-8 occurred in both model systems, the presence of macrophages was a determining factor of the IL-8 release profile after AMBR2 treatment. In dual co-culture systems, the concentration of IL-8 was significantly higher (K–W) than in triple co-culture systems, *p* = 0.00035) at 48 h, while this effect disappeared at 72 h (K–W). The donor-derived polymicrobial background was a less determining factor and only resulted in a significant difference in dual co-cultures treated with *L. casei* AMBR2 72 h after the start of the experiment (K–W, post hoc non-significant).

IL-10 concentrations in the basolateral medium were below the quantification limit, except in co-cultures containing macrophages after 72 h of co-culture (Fig. 8c). In co-cultures including donor-derived microbial communities, treatment with *L. casei* AMBR2 increased IL-10 release in a noticeable, yet non-significant manner, whereas there was no difference between the sterile control group and cell layers treated with AMBR2 alone.

TNF-*α* release in the basolateral compartment was below the quantification limit for all groups on time points 0–48 h; therefore, only the 72 h time point is shown (Fig. 8d). After 72 h of co-culture, set-ups containing macrophage-like cells had higher concentrations of TNF-*α* than set-ups without macrophage-like cells (*p* = 0.014, K–W). Presence of *L. casei* AMBR2 elicited an increase in TNF-*α* release in triple co-culture (*p* = 0.038, K–W), independent of the donor background. In dual co-culture, this was observed as well (*p* = 0.0003, K–W), however, further inspection of the data showed that TNF-*α* release was only above quantification limit in cell layers treated with AMBR2 alone.

#### Bacterial growth and phenotypic diversity

**Figure 9:**
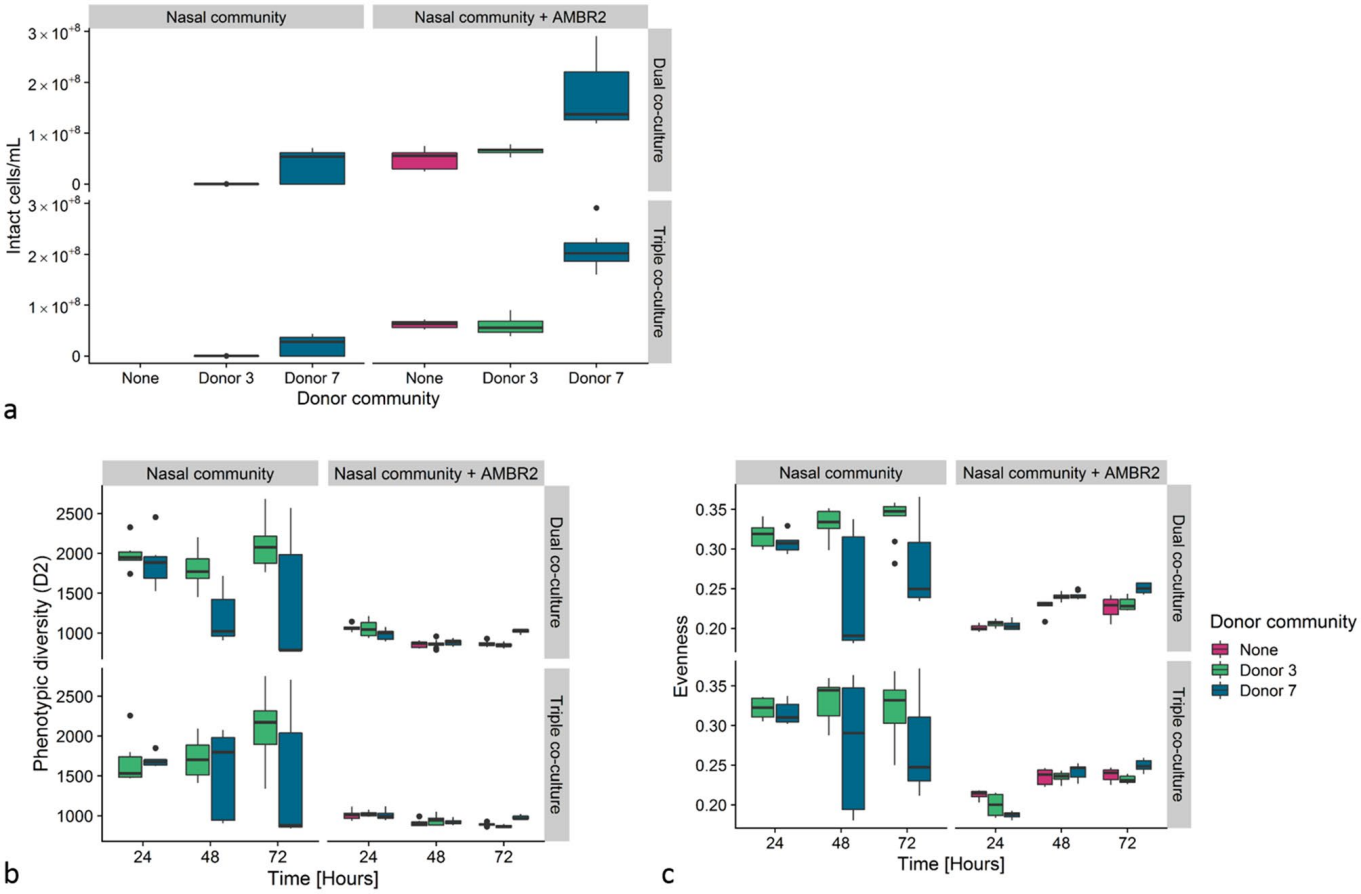
Intact cell density in apical washing fluid 72 h after inoculation of *L. casei* AMBR2 in dual and triple co-culture model systems with nasal microbiota (**a**). Phenotypic alpha diversity (D2) (**b**) and (**c**) phenotypic evenness of bacterial communities with and/or without *L. casei* AMBR2 present on epithelial cell layers in dual and triple co-culture, based on flow cytometry data. “None” indicates the absence of a donor-derived polymicrobial background.

Intact bacterial cell density in the apical washing fluid 72 h after the start of the experiment is displayed in Fig. 9a. *L. casei* AMBR2 displayed presence and growth on all cell layers on which it was inoculated (5.0 × 10^7^ ± 1.9 × 10^7^ intact cells mL^−1^ in dual co-culture, 6.2 × 10^7^ ± 6.7 × 10^7^ intact cells mL−^−1^ in triple co-culture, average ± standard deviation). Nasal bacteria derived from donor 3 showed limited growth in dual (1.7 × 10^5^ ± 1.7 × 10^5^ intact cells mL^−1^) and triple co-culture set-ups (2.1 × 10^5^ ± 1.0 × 10^5^ intact cells mL^−1^), contrarily to those derived from donor 7, which were able to grow in both model systems (4.0 × 10^7^ ± 3.0 × 10^7^ intact cells mL^−1^ in dual co-culture, 2.3 × 10^7^ ± 1.8 × 10^7^ intact cells mL^−1^ in triple co-culture) to similar cell densities as *L. casei* AMBR2 alone. In communities where AMBR2 was inoculated in a background of donor 7, cell densities were higher than the donor or AMBR2 alone, indicating growth of both inocula or a synergistic effect between the two (1.7 × 10^8^ ± 6.1 × 10^7^ intact cells mL^−1^ in dual co-culture, 2.1 × 10^8^ ± 3.7 × 10^7^ intact cells mL^−1^ in triple co-culture). The presence of macrophages did not significantly affect the intact cell density, except for donor 3 with AMBR2, where the intact cell count was lower in triple co-culture (Wilcoxon test, *p* = 0.022).

The phenotypic alpha diversity (D2) is presented in Fig. 9b. For donor 3, phenotypic alpha diversity in both model systems was decreased in presence of *L. casei* AMBR2 (K–W, *p* < 0.001, followed by Dunn’s test, *p* < 0.001), whereas the type of model system did not significantly affect phenotypic diversity with or without AMBR2. Phenotypic diversity decreased over time in donor 3 communities with AMBR2 (K–W, *p* < 0.001, Dunn’s post hoc, *p* < 0.05), as well as donor 3 communities without AMBR2 (K–W, *p* = 0.012, Dunn’s post hoc, *p* = 0.01 between 48 and 72 h). For donor 7, large variation in phenotypic diversity was observed between time points and samples in communities in which AMBR2 was not introduced and there was no significant decrease in phenotypic diversity over time. In both dual and triple co-culture, a decrease upon introduction of AMBR2 in these communities occurred from 24 to 48 h, whereas after 72 h phenotypic diversity had increased again to the level it was at 24 h (K–W, *p* < 0.001, Dunn’s post hoc, *p* < 0.001).

We therefore assessed whether loss of phenotypic evenness was a driver for phenotypic diversity loss upon introduction of AMBR2 (Fig. 9c). Without AMBR2, donor 3 had a more even community than donor 7 in dual (Wilcoxon test, *p* < 0.01) and triple co-culture systems (Wilcoxon test, *p* < 0.01), whereas with AMBR2, donor 7 had a more even community than donor 3 with AMBR2 (Wilcoxon test, *p* = 0.02) in co-cultures without macrophage-like cells, as well as in co-cultures with macrophage-like cells (Wilcoxon test, *p* = 0.01). Donor communities in which AMBR2 was introduced had a significantly lower evenness than communities consisting of donor microbiota alone (K–W, *p* < 0.001), regardless of donor or model system.

## Discussion

We examined the ability of the potential live biotherapeutic *L. casei* AMBR2 to grow on and attach to respiratory epithelial cell layers in the presence of donor-derived nasal microbiota from 10 different healthy donors. After the selection of two of these donors, the host–microbe interaction of AMBR2 was tested in more physiologically relevant host–microbe interaction models in the polymicrobial background of these donors.

During the donor screening, we observed that AMBR2 could adhere and survive or grow in all donor backgrounds, albeit with interindividual differences. Phylogenetic community analysis showed that all of our donors belonged to the intermixed *Staphylococcus–Corynebacterium–Dolosigranulum* diverse profile^66^, which represented 91% of the participants (n = 90) in a Belgian-Dutch cohort. They also belonged to the most prevalent nasal community state types found by Liu et al.^51^ in a Danish cohort and by Lehtinen et al.^67^ in a U.S. American cohort. Incubation of these nasal bacterial communities on submerged Calu-3 respiratory cell layers resulted in outgrowth of either *Staphylococcus* sp. (without AMBR2) or *Lactobacillus* sp. (with AMBR2), with the exception of donor 3, which had a community dominated by unclassified *Enterobacteriaceae*, regardless of presence of AMBR2, after 24 h. In our, and other similar, more complex model systems^57,58^, a consistent increase of *Staphylococcus* sp. in co-cultured communities compared to the original inoculum has been observed. The growth of *Staphylococcus* community members was favoured as well here in this simplified submerged monolayer set-up when no additional lactobacilli were introduced. We observed a negative correlation between the relative abundance of *Staphylococcus* spp. in the inoculum and the relative abundance of *Lactobacillus* spp. 24 h after introduction of *L. casei* AMBR2 (partial 16S rRNA gene sequencing). However, in 8 out of 9 of these communities *Lactobacillus* sp. relatively represented > 75% of the community as determined by partial 16S rRNA gene sequencing and flow cytometry. Furthermore, through selective plating and flow cytometry, we detected increased lactic acid bacteria and predicted AMBR2 cell counts after 24 h compared to the inoculum, indicating growth in 8 out of 10 donor backgrounds. This colonisation success can partly be explained by the high inoculation density of AMBR2 relative to the nasal microbiota, as it has been observed that absolute abundance is a driving factor in nasal bacterial interactions^51^.

Most striking was the bacterial community of donor 3, who had relative abundances of > 90% of an OTU from the genus *Enterobacteriaceae* sp., presumably *Klebsiella aerogenes* (eHOMD^54^), with or without introduction of AMBR2. This OTU was present in other nasal communities, yet no overgrowth was observed in other donors and no conclusions could be made on whether this was the same species or strain. *Klebsiella aerogenes*, previously known as *Enterobacter aerogenes*, is an opportunistic pathogen, which had a prevalence of 2.6% in a healthy German study population (n = 1878)^68^. Interestingly, they showed that significant differences in the co-colonisation pattern of *S. aureus* and enterobacteria existed, with individuals being colonised with *S. aureus* having less frequent colonisation of *Enterobacteriaceae*. Donor 3 was characterised by lower intact bacterial cell counts in their nasal swab than the other donors (10^4^ cells mL^−1^ vs. between 10^5^ and 10^6^ cells mL^−1^ for all other donors); the fact that the inocula of other donors required more dilution at the start of the experiment, could have stochastically decreased the relative abundance of *Enterobacteriaceae* and thus have reduced the potential for *Enterobacteriaceae* sp. overgrowth in the other donors in which it occurred.

The nasal communities included in our screening assay represented the most commonly observed community types in the nose^51,66,67^. Future studies would benefit from including individuals with other community types, such as those dominated by e.g.* Moraxella* or *Enterobacteriaceae*, and a broader age range and background to better reflect the general population and elucidate bacterial community patterns associated with degree of colonisation permissiveness.

When applied in dual and triple co-culture systems, we observed that nor the donor communities, nor AMBR2, induced a cytotoxic response during the course of the experiment. In presence of a polymicrobial background, *L. casei* AMBR2 increased the epithelial resistance of differentiated epithelial cell layers, whereas this did not occur without donor-derived microbiota. The presence of donor-derived bacteria without *L. casei* AMBR2 resulted in a slight decrease in epithelial resistance, which was restored through the introduction of AMBR2. TLR-2 (Toll-like Receptor-2) ligands peptidoglycan and the synthetic bacterial lipopeptide Pam3CSK4 were previously found to increase epithelial barrier functionality in a Calu-3 cell-based setup through upregulation of tight junction proteins claudin-1 and zonula occludens 1^69^. Martens and colleagues (submitted, 2020) have demonstrated that *L. casei* AMBR2 could increase epithelial barrier function in primary cell layers derived from CRSwNP (CRS with nasal polyps) patients via upregulation of the same tight junction proteins, presumably through a TLR-2 mediated mechanism. This mechanism remains however to be substantiated, as well as the responsible microbe associated molecular patterns associated with TLR2.

Throughout the experiment, pro-inflammatory IL-8 release remained in the range of sterile controls in similar set-ups^70,71^, or on Calu-3 monolayers treated with AMBR2^41^, and was six- to tenfold lower than a similar set-up treated with 10^7^ *Staphylococcus aureus* cells mL^−1^^71^. The introduction of AMBR2 in the dual and triple co-culture systems elicited an increase in IL-8 release. We observed a difference in IL-8 release profile dependent on the presence or absence of THP-1 derived macrophages. Without THP-1 derived macrophages, a peak in IL-8 release was observed after 48 h, whereas this did not occur in the triple co-culture systems containing THP-1-derived macrophages. Several probiotic lactobacilli, living or heat-killed, can also induce IL-10 release in THP-1 derived macrophages in direct contact^72,73^. We observed an increase in IL-10 release in triple co-culture systems, albeit in 100-fold lower concentrations than observed by Rocha-Ramirez et al.^72^, possibly due to the lack of direct contact between macrophages and bacterial cell wall compounds, and their use of primary human monocyte derived macrophages instead of the THP-1 cell line. In mice, remote induction of IL-10 by *Lactobacillus plantarum*, reclassified as *Lactiplantibacillus plantarum*^42^ in the gut could reduce the induction of IL-8 after nasal infection with *Klebsiella pneumoniae*^74^, a mechanism which might possibly explain the reduced IL-8 release in our triple co-culture model system. This underlines the importance of incorporating immune cells as well as epithelial cells to study host–microbe interactions in the URT.

*L. casei* AMBR2 could grow in model systems based on differentiated airway epithelial structures, regardless of the presence of natural nasal microbiota or macrophages. Regrowth occurred after daily washes to mimic the nasal fluid flow, indicating adherence of AMBR2 to the respiratory cell layer. While AMBR2 colonisation in the microbial background of donor 3 was reduced compared to AMBR2 alone or in presence of donor 7 in the simplified screening set-up, this did not occur in the more complex dual and triple co-culture set-ups, despite a relative abundance of 0.29% unclassified *Enterobacteriaceae* in the inoculum of donor 3. De Boeck et al.^41^ showed that AMBR2 has superior adaptive properties for survival in the URT, such as fimbriae for attachment, and the ability to cope with oxidative stress through the presence of catalase genes. The dual and triple co-culture set-ups mimic the microenvironment of sinonasal cavities more closely than a submerged monolayer in terms of nutrient availability^75^, host response^76^, and growth surface^61,62^. Liu et al.^6^ demonstrated that *S. epidermidis* could exclude opportunistic pathogens through host-mediated competition by the induction of antimicrobial peptides. Furthermore, *L. casei* AMBR2 has antimicrobial activity against staphylococci and *Enterobacteriaceae*^41^. These factors may explain the lack of *Enterobacteriaceae* overgrowth in dual and triple co-culture systems inoculated with nasal bacteria from donor 3 compared to the submerged monolayer co-culture, as well as a similar growth of AMBR2 in donor backgrounds in these more relevant model systems.

Major limitations of this study are the use of cancer cell lines, the limited number of cell types tested, and the low number of individuals used for nasal microbiota sampling. A first major disadvantage of our study is the small number of individuals used for nasal microbiota sampling, their narrow age range, the homogeneity of their environment, and their high interindividual variability. We did not fully cover the variability observed in the population, therefore further validation studies require a larger and more heterogeneous sample group. Next to this, the incorporation of the microbiota of a larger and more heterogeneous sample cohort in our model system can result in improved understanding of the mechanisms driving bacterial community evolution upon introduction in the in vitro environment of the model system and elucidate possible differences between community types. Due to the existence of bacterial community state types, a preselection of this sample cohort would be appropriate in order to cover sufficient variability.

Our screening and colonisation assays were performed using nasal microbiota obtained from clinically healthy individuals. In order to assess the factors driving colonisation potential and probiotic potency in a polymicrobial background more representative of the CRS environment, CRS patient-derived sinonasal communities should be included, stratified according to CRS sub- or endotypes^10,26,77^. Furthermore, the respiratory epithelial dual and triple co-culture systems are based on non-inflamed epithelial cell layers with intact tight junctions. A dysfunctional epithelial barrier is characteristic for CRSwNP sinonasal epithelium^78–80^, not only resulting in increased potential of microorganisms crossing the epithelial barrier, but also leakage of nutrients into the sinonasal cavity^75^. Furthermore, mucus production and oxygen concentrations can be affected in CRS, potentially steering the sinonasal microbiota towards dysbiosis^81–83^. Mechanistic studies of probiotic potency should therefore not only include CRS-patient derived microbiota, but also an epithelial microenvironment mimicking that found in CRS.

Our goal was to develop a co-culture model system for the human upper respiratory tract with relevant readouts for responses to microbial colonisation and inflammation. We have chosen cancer cell lines for their reproducible behaviour, as a generic model system where the interindividual variability only originates from the bacterial community, rather than from both host and bacterial model constituents. We have chosen the Calu-3 cell line for its potential to be differentiated at air–liquid interface, mucin production, and establishment of tight junctions. Next to this, the behaviour of this cell line is well characterised^59–62,84^. Other (nasal) respiratory cell lines have limitations for use in this type of model system due to forming leaky epithelium and/or not differentiating at ALI, for example NuLi-1 cells^85^, which is not representative for healthy nasal epithelium. BEAS-2B cells, a human bronchial epithelial cell line, are frequently used in URT research, fail to establish tight junctions and do not form pseudostratified epithelium upon differentiation, both of which are essential in the sinonasal setting^60^. 16HBE cells, another human bronchial epithelial cell line, form tight junctions, yet have lower mucin production than their Calu-3 counterpart, rendering these cells suboptimal to establish a sinonasal model system^86^. It should however be noted that Calu-3 cells are limited in reflecting the responses to inflammatory stimulation observed in primary cells^70^. Recently, advances have been made in the immortalisation and differentiation of primary sinonasal epithelial cells^57^ and in the generation of upper respiratory tract cells from pluripotent stem cells^87^. These cell types can be more representative than the cancer cell line used in this model system.

## Conclusion

*L. casei* AMBR2, a potential live biotherapeutic for the human upper respiratory tract, is able to attach to and survive on a respiratory epithelial monolayer in the presence of donor-derived nasal microbiota, representative for the most prevalent community type in a healthy population. While attachment and survival of AMBR2 occurred in all donor-derived communities, its growth was severely inhibited in one case of *Enterobacteriaceae* overgrowth. This type of overgrowth did not occur in differentiated respiratory epithelial cell layers at the air–liquid interface, emphasising the importance of employing physiologically representative model systems in host–microbe interaction research. Introduction of AMBR2 in dual and triple co-culture systems did not induce cytotoxicity and could inhibit the reduction in epithelial barrier function caused by the nasal community. Inclusion of macrophage-like cells showed that introduction of AMBR2 could restore the release of the anti-inflammatory cytokine IL-10 in a polymicrobial background. This underlines the potential potency of *L. casei* AMBR2 as a live biotherapeutic product in the polymicrobial background in which it will be introduced.

## Materials and methods

### Human cell culture

Calu-3 (ATCC HTB-55, Lot.: 62657853, ATCC, LGC Standards, Molsheim, France) cells were cultured at 37 °C, 10% CO_2_ and 90% relative humidity in Minimal Essential Medium (MEM, Gibco, Sigma-Aldrich, Overijse, Belgium) supplemented with 10% (v/v) heat inactivated fetal bovine serum (FBSi, BioWest Premium, Lot.: S18407S1810, VWR, Oud-Heverlee, Belgium). For the donor screening assay, cells were used at passage 9 counted from the original vial. An antibiotic/antimycotic solution of streptomycin-penicillin-amphothericin B (Antibiotic Antimycotic Solution 100 ×, Sigma-Aldrich) was added (1% v/v) to the growth medium for routine culture. Prior to seeding in 24-well plates (Tissue Culture Treated, Non-coated, Corning, Sigma-Aldrich), cells were stained with Trypan blue (0.4% w/v in phosphate buffered saline (PBS), Sigma-Aldrich) to assess viability and counted using a Neubauer counting chamber. Cells were seeded at density of 3.95 × 10^4^ cells cm^−2^, and maintained until confluent. 7 days prior to the bacterial inoculation the medium was switched to FBSi supplemented medium without antibiotic/antimycotic solution.

In this assay, dual and triple co-culture models were used^58^. In the dual co-culture model, Calu-3 cells were combined with bacteria, while in the triple co-culture model, Calu-3 cells were combined with bacteria and macrophage-like cells. Calu-3 cells (passage 9) were seeded at a density of 10^5^ cells cm^−2^ in 6-well Transwell inserts (4.67 cm^2^ culture surface, Corning, Sigma-Aldrich), and maintained under submerged conditions until fully confluent. When confluent, the cell layers were brought to air–liquid interface (ALI) for differentiation for 21 days. 7 days prior to the bacterial inoculation the medium was switched to FBSi supplemented medium without antibiotic/antimycotic solution. THP-1 cells (ATCC TIB-202, passage 43–50) were seeded in 6-well plates at a density of 10^5^ cells/well in RPMI 1640 medium with Glutamax, supplemented with 10% FBSi. Monocytes were differentiated into macrophages using phorbol-12-myristate-12-acetate (PMA, Sigma-Aldrich) at a concentration of 100 nM for 72 h. THP-1 derived macrophages and Calu-3 cell layers were combined prior to bacterial inoculation by placing the inserts with differentiated Calu-3 cell layers in the 6-well plate containing THP-1 derived macrophages.

### Donor recruitment and sampling

Nasal swabs were collected from 10 different healthy donors, aged between 24 and 32 (27 ± 2.5 years) and 80% female/20% male). Donor characteristics are included in Supplementary Table 1. Inclusion criteria were: clinical health, never-smoker, aged between 18 and 65, no asthma or atopy, no antibiotic use in the past 4 weeks, no use of topical corticosteroids in the past 4 weeks and no history of chronic or recurrent respiratory illnesses such as (chronic) rhinosinusitis, chronic obstructive pulmonary disease or aspirin exacerbated respiratory disease. Research incubation work with nasal microbiota from human origin was approved by the ethical committee of the Ghent University hospital under registration number B670201214538 and the research was performed in accordance with the relevant guidelines and regulations. Informed consent was obtained from all participants. Two nasal swabs (Flocked Swab, sterile, Copan, VWR, Oud-Heverlee, Belgium) were taken from the posterior nose and care was taken to avoid contamination from the nostrils. One swab was stored at − 20 °C until further analysis. The other swab tip was placed in an Eppendorf tube in sterile phosphate buffered saline (PBS) and placed on a Vortex shaker during 60 s. The tip was then removed from the tube and dilutions were prepared for flow cytometry and inoculation of the respiratory tract cell layers.

For dual and triple co-culture, two donors were selected from the donor pool of the screening assay, based on the ability of *L. casei* AMBR2 to grow in their microbial background. Donor 3 and donor 7 were used, since they displayed respectively the lowest and the highest growth of *L. casei* AMBR2 in the polymicrobial background of their nasal swab. Sampling was performed as described earlier.

### Bacterial culture

*L. casei* AMBR2^41^ was retrieved from a − 80 °C glycerol stock and transferred to MRS agar (De Man, Rogosa, and Sharpe agar, Carl Roth, Karlsruhe, Germany), 37 °C. A colony was picked and transferred to MRS broth (Carl Roth), 37 °C for overnight incubation. Prior to determination of the concentration using flow cytometry, and inoculation on the cell layers, the bacterial culture was washed twice (5 min at 5000×*g*) using sterile PBS, and afterwards suspended in sterile PBS.

### Colonisation of *L. casei* AMBR2 in upper respiratory tract host–microbe interaction set-ups

#### Donor screening assay

For each donor (n = 10), Calu-3 layers were inoculated with donor microbiota only (n = 3), or with donor microbiota and *L. casei* AMBR2 (biological replicates, n = 3). Sterile controls (n = 3), and *L. casei* AMBR2 only (n = 3) wells were included. First, a suspension of 10^3^ intact nasal bacterial cells in PBS was added to the Calu-3 cell layer. Two hours after the inoculation of nasal bacteria, 100 µL of 10^8^ intact cells mL^−1^ PBS of *L. casei* AMBR2 was introduced in the nasal community. In sterile wells, or wells with donor communities, but without *L. casei* AMBR2, an equal amount of PBS was added. The cell layers with microbial communities were incubated for 24 h at 37 °C, 10% CO_2_, and 90% relative humidity. Bacteria that were not strongly adhered to the Calu-3 cell layer were sampled by pipetting up and down and removing the medium from the cell layers, and gently pipetting sterile PBS to remove the remaining non-adhered bacteria in an additional washing step. The cell layers were then incubated with 0.2% v/v Triton X-100 in PBS to detach the bacteria that were bound to the cell layers (adhered bacteria). Samples were taken for flow cytometry, DNA extraction, and plating on MRS agar. Microbial suspensions that were plated after sampling from the epithelial cell layers, were first diluted in sterile PBS, then plated on MRS agar and incubated at 37 °C for 48 h.

#### Colonisation of *L. casei* AMBR2 in dual and triple co-culture set-ups

In this assay, a comparison was made between the colonisation of *L. casei* AMBR2 in dual (Calu-3 + microbiota) and triple co-culture (Calu-3 + THP-1 + microbiota) set-ups inoculated with nasal bacteria from identical donors as described in De Rudder et al.^58^. Nasal microbiota samples were obtained from two healthy donors selected from the donor screening as earlier described, and for each donor we inoculated Calu-3 cell layers in co-culture with THP-1 cells (n = 6) and Calu-3 cell layers without THP-1 cells (n = 6) with 100 µL of 10^4^ intact cells mL^−1^. Two hours after the inoculation of nasal bacteria, 100 µL of 10^9^ intact cells mL^−1^ PBS of *L. casei* AMBR2 was introduced in half of the nasal communities in dual (n = 3) or triple (n = 3) co-culture models. Sham inoculated cell layers (n = 3), and *L. casei* AMBR2 only inoculated cell layers (n = 3) were included for dual and triple co-culture set-ups.

#### Cytotoxicity

Cytotoxicity of the epithelial layers was assessed by quantifying the lactate dehydrogenase (LDH) leaked from the cell layer in the basolateral compartment using the Pierce LDH Cytotoxicity Assay Kit (Thermo Fisher Scientific, Merelbeke, Belgium) according to the manufacturer’s instructions, using an Infinite M200 Pro platereader (Tecan). This assay was chosen because no bacteria (which often express LDH that can cross-react) are present in the basolateral compartment.

#### Transepithelial electrical resistance

The transepithelial electrical resistance (TEER) of the cell layers was assessed using an epithelial Volt-Ohmmeter (MilliCell ERS-2, Millipore, Merck, Overijse, Belgium) with an MERSSTX01 electrode. TEER measurements were performed twice per insert to account for electrode variability. The measured TEER values were corrected by subtracting the TEER value of a Transwell insert without cells under identical conditions and are given in Ω. TEER values were measured during the co-culture experiment with 2.5 mL cell culture medium in the basolateral compartment and 1 mL PBS in the apical compartment. The chopstick electrode was sterilized with 70 v% ethanol in water and rinsed with cell culture medium after each insert measurement to avoid cross-contamination.

#### Cytokine secretion

Secretion of cytokines IL-8, TNF-α, and IL-10 into the basolateral compartment was measured with an enzyme linked immunosorbent assay (ELISA) at t = 0 h, 24 h, 48, and 72 h after inoculation. IL-8 and IL-10 were measured with Human Uncoated ELISA kits (Invitrogen, Merelbeke, Belgium) according to the manufacturer’s instructions. TNF-*α* was measured with an eBioscience Human TNF alpha ELISA Ready-SET-Go! Kit (Invitrogen) according to the manufacturer’s instructions. Colour development was measured with an Infinite M200 Pro platereader (Tecan).

### Intact/damaged staining and flow cytometry

Intact bacterial cell densities were measured using flow cytometry, through SYBR Green/propidium iodide staining (SGPI), as described in Van Nevel et al.^88^. Samples were incubated for 13 min at 37 °C for SGPI staining. Flow cytometric analysis of the microbial cells present in the suspension was performed using a C6 + Accuri flow cytometer (BD Biosciences, Erembodegem, Belgium) equipped with a 488 nm laser, following previously described methods^89^. Fluorescence events were monitored using the fluorescence detector (FL)1 533/30 nm and FL3 > 670 nm optical detectors. Forward and sideways-scattered light was collected as well. A threshold value of 1000 was applied on the FL1 channel. Data for the donor screening experiment are available on Flow Repository (FR-FCM-Z2JS).

### Flow cytometric fingerprinting and classification of populations

Flow cytometric fingerprints were calculated using the R package Phenoflow^90^. Fingerprints were constructed based on forward scatter, sideward scatter, SG fluorescence and PI fluorescence of the sample using 128 bins per channel (nbin = 128) and a bandwidth of 0.01 (bw = 0.01) for the kernel density estimator calculated at each bin. Fingerprint-based ecological parameters (alpha- and beta-diversity, Pareto-evenness) were calculated using the *Diversity rf()* function, the *beta div fcm()* function and the *Evenness()* function from the R package Phenoflow^90^. Phenotypic beta-diversity was calculated using Bray–Curtis distances. Principal Coordinates Analysis (PCoA) and non-metric multidimensional scaling (NMDS) was used to create ordination plots of the beta-diversity.

To estimate the intact cell density of donor-derived bacteria and *L. casei* AMBR2 in wells containing a combination of both, Random Forest classifiers were created using *RandomF FCS()*, with tenfold cross-validation (repeated 3 times). These classifiers were then applied to predict the intact cell counts of AMBR2 and donor bacteria in a mixed sample using *RandomF predict()*, both from the R package Phenoflow^90^. Models were trained and tested with flow cytometry data of *L. casei* AMBR2 alone and the respective donor community alone on 2000 events for the non-adhered communities (75% training, 25% testing). In samples with low bacterial cell densities, less events were used (Table S1—part 1). Accuracy and Cohen’s Kappa statistic were evaluated for each individual model, and classifiers with accuracy ≥ 90% and a Kappa value ≥ 85% were used to estimate the events originating from either the donor microbiota or *L. casei* AMBR2. Accuracy and Kappa values for each classifier are presented in Table S2—part 1. Adhered communities had lower cell densities than non-adhered communities, therefore between 20 and 200 events could be used to train and test the classifiers (75% training, 25% testing). Accuracy and Cohen’s Kappa statistic were evaluated for each individual model, and classifiers with accuracy ≥ 80% and a Kappa value ≥ 60% were used to estimate the events originating from either the donor microbiota or *L. casei* AMBR2 (Table S2—part 2).

### DNA extraction, partial 16S rRNA gene sequencing and data analysis

DNA was extracted from microbial communities as described in Vilchez-Vargas et al.^91^. Sequencing of the V3–V4 region of the 16S rRNA gene with forward primer (341F) and reverse primer (785R) was done on an Illumina MiSeq in PE 2X300 bp using V3 chemistry (Illumina) by Biofidal (Vaulx-en-Velin, France—https://www.biofidal-lab.com), as described in Supplementary Method (6).

The amplicon data were processed as described in^92^ using mothur (v.1.39.5), largely based on the protocol by the Schloss lab^93,94^ and described in more detail in Supplementary Method (6). Richness was estimated using the Chao1 index^95^ and diversity was estimated the Inverse Simpson index. Samples from the dual and triple co-culture set-ups yielded insufficient biomass for sequencing, therefore only the swab samples (t = 0 h, inoculum) are displayed. Data are available under the BioProject accession number PRJNA627613.

### Statistical analysis

All statistical analyses were performed and graphs were created using R version 3.6.1 (The R Foundation for Statistical Computing). The normality of the data was assessed using the Shapiro–Wilk test, and homoscedasticity was assessed with the Bartlett test for equal variances. In case normality and homoscedasticity were rejected, the Kruskal–Wallis test (K–W) was used for multiple comparisons, followed by Dunn’s post hoc test with Holm’s correction or Wilcoxon Ranked Sum tests. When normality and homoscedasticity were not rejected, two-way ANOVA was performed, followed by Tukey’s HSD post hoc test. Significance was considered at the 5% level (*α* = 0.05).

### Additional information

.

## Supplementary information

Supplementary Information.

## References

[1] Khan R, Petersen FC, Shekhar S (2019). Commensal bacteria: an emerging player in defense against respiratory pathogens. Front. Immunol..

[2] Dumas A (2018). The host microbiota contributes to early protection against lung colonization by mycobacterium tuberculosis. Front. Immunol..

[3] Bomar L, Brugger SD, Yost BH, Davies SS, Lemon KP (2016). *Corynebacterium accolens* releases antipneumococcal free fatty acids from human nostril and skin surface triacylglycerols. Mbio.

[4] Belkaid Y, Hand TW (2014). Role of the microbiota in immunity and inflammation. Cell.

[5] Jain R, Waldvogel-Thurlow S, Darveau R, Douglas R (2016). Differences in the paranasal sinuses between germ-free and pathogen-free mice. Int. Forum Allergy Rhinol..

[6] Liu Q (2020). *Staphylococcus epidermidis* contributes to healthy maturation of the nasal microbiome by stimulating antimicrobial peptide production. Cell Host Microbe.

[7] Salzano FA (2018). Microbiota composition and the integration of exogenous and endogenous signals in reactive nasal inflammation. J. Immunol. Res..

[8] Hoggard M (2017). Chronic rhinosinusitis and the evolving understanding of microbial ecology in chronic inflammatory mucosal disease. Clin. Microbiol. Rev..

[9] Hoggard M (2017). Evidence of microbiota dysbiosis in chronic rhinosinusitis. Int. Forum Allergy Rhinol..

[10] Lee K, Pletcher SD, Lynch SV, Goldberg AN, Cope EK (2018). Heterogeneity of microbiota dysbiosis in chronic rhinosinusitis: potential clinical implications and microbial community mechanisms contributing to sinonasal inflammation. Front. Cell. Infect. Microbiol..

[11] Mackenzie BW (2017). Bacterial community collapse: a meta-analysis of the sinonasal microbiota in chronic rhinosinusitis. Environ. Microbiol..

[12] Piters WAAD, Sanders EAM, Bogaert D (2015). The role of the local microbial ecosystem in respiratory health and disease. Philos. Trans. R. Soc. B Biol. Sci..

[13] Wilson MT, Hamilos DL (2014). The nasal and sinus microbiome in health and disease. Curr Allergy Asthma Rep..

[14] de Loos DD (2019). Prevalence of chronic rhinosinusitis in the general population based on sinus radiology and symptomatology. J. Allergy Clin. Immunol..

[15] Hastan D (2011). Chronic rhinosinusitis in europe - an underestimated disease. A ga(2)len study. Allergy.

[16] Fokkens W (2020). European position paper on rhinosinusitis and nasal polyps 2020. Rhinol. J..

[17] Fokkens WJ (2012). Epos 2012: European position paper on rhinosinusitis and nasal polyps 2012. A summary for otorhinolaryngologists. Rhinology.

[18] Lam K, Schleimer R, Kern RC (2015). The etiology and pathogenesis of chronic rhinosinusitis: a review of current hypotheses. Curr. Allergy Asthma Rep..

[19] Akdis CA (2013). Endotypes and phenotypes of chronic rhinosinusitis: a practall document of the European academy of allergy and clinical immunology and the American academy of allergy, asthma and immunology. J. Allergy Clin. Immunol..

[20] Hellings PW (2017). Positioning the principles of precision medicine in care pathways for allergic rhinitis and chronic rhinosinusitis—a EUFOREA-ARIA-EPOS-AIRWAYS icp statement. Allergy.

[21] Walker A, Philpott C, Hopkins C (2019). What is the most appropriate treatment for chronic rhinosinusitis?. Postgrad. Med. J..

[22] Head K (2016). Systemic and topical antibiotics for chronic rhinosinusitis. Cochrane Database Syst. Rev..

[23] Hoggard M (2018). Inflammatory endotypes and microbial associations in chronic rhinosinusitis. Front. Immunol..

[24] Aurora R (2013). Contrasting the microbiomes from healthy volunteers and patients with chronic rhinosinusitis. JAMA Otolaryngol. Head Neck Surg..

[25] Choi EB (2014). Decreased diversity of nasal microbiota and their secreted extracellular vesicles in patients with chronic rhinosinusitis based on a metagenomic analysis. Allergy.

[26] Cope EK, Goldberg AN, Pletcher SD, Lynch SV (2017). Compositionally and functionally distinct sinus microbiota in chronic rhinosinusitis patients have immunological and clinically divergent consequences. Microbiome.

[27] Abreu NA (2012). Sinus microbiome diversity depletion and *Corynebacterium tuberculostearicum* enrichment mediates rhinosinusitis. Sci. Transl. Med..

[28] Man WH, Piters WAAD, Bogaert D (2017). The microbiota of the respiratory tract: gatekeeper to respiratory health. Nat. Rev. Microbiol..

[29] De Boeck I (2019). Anterior nares diversity and pathobionts represent sinus microbiome in chronic rhinosinusitis. Msphere..

[30] Krysko O (2011). Alternatively activated macrophages and impaired phagocytosis of saureus in chronic rhinosinusitis. Allergy.

[31] Bardy JJ (2018). *Staphylococcus aureus* from patients with chronic rhinosinusitis show minimal genetic association between polyp and non-polyp phenotypes. BMC Ear Nose Throat Disord..

[32] Cope EK, Goldberg AN, Pletcher SD, Lynch SV (2016). A chronic rhinosinusitis-derived isolate of *Pseudomonas aeruginosa* induces acute and pervasive effects on the murine upper airway microbiome and host immune response. Int. Forum Allergy Rhinol..

[33] Cope EK, Lynch SV (2015). Novel microbiome-based therapeutics for chronic rhinosinusitis. Curr. Allergy Asthma Rep..

[34] Martens K (2018). Probiotics for the airways: Potential to improve epithelial and immune homeostasis. Allergy.

[35] O’Toole PW, Marchesi JR, Hill C (2017). Next-generation probiotics: the spectrum from probiotics to live biotherapeutics. Nat. Microbiol..

[36] Popova M (2012). Beneficial effects of probiotics in upper respiratory tract infections and their mechanical actions to antagonize pathogens. J. Appl. Microbiol..

[37] Choi SP (2018). Oral administration of *Lactobacillus plantarum* cjlp133 and cjlp243 alleviates birch pollen-induced allergic rhinitis in mice. J. Appl. Microbiol..

[38] Martensson A (2017). Clinical efficacy of a topical lactic acid bacterial microbiome in chronic rhinosinusitis: A randomized controlled trial. Laryngoscope Investig. Otolaryngol..

[39] De Grandi R (2019). Putative microbial population shifts attributable to nasal administration of *Streptococcus salivarius* 24smbc and *Streptococcus oralis* 89a. Probiotics Antimicrob. Proteins.

[40] Bidossi A (2018). Probiotics *Streptococcus salivarius* 24smb and *Streptococcus oralis* 89a interfere with biofilm formation of pathogens of the upper respiratory tract. BMC Infect. Dis..

[41] De Boeck I (2020). Lactobacilli have a niche in the human nose. Cell Rep..

[42] Zheng J (2020). A taxonomic note on the genus lactobacillus: Description of 23 novel genera, emended description of the genus *Lactobacillus**beijerinck* 1901, and union of lactobacillaceae and leuconostocaceae. Int. J. Syst. Evol. Microbiol..

[43] Schwartz J (2016). Topical probiotics as a therapeutic alternative for chronic rhinosinusitis: a preclinical proof of concept. Am. J. Rhinol. Allergy.

[44] van den Broek MFL, De Boeck I, Claes IJJ, Nizet V, Lebeer S (2018). Multifactorial inhibition of lactobacilli against the respiratory tract pathogen *Moraxella catarrhalis*. Benef. Microbes.

[45] Dunne EM (2014). Investigating the effects of probiotics on pneumococcal colonization using an in vitro adherence assay. J. Vis. Exp..

[46] Walter J, Maldonado-Gómez M, Martníez I (2017). To engraft or not to engraft: an ecological framework for gut microbiome modulation with live microbes. Curr. Opin. Biotechnol..

[47] Allen EK (2014). Characterization of the nasopharyngeal microbiota in health and during rhinovirus challenge. Microbiome.

[48] Bassiouni A, Cleland EJ, Psaltis AJ, Vreugde S, Wormald PJ (2015). Sinonasal microbiome sampling: a comparison of techniques. PLoS ONE.

[49] Bassis CM, Tang AL, Young VB, Pynnonen MA (2014). The nasal cavity microbiota of healthy adults. Microbiome.

[50] Biswas K, Hoggard M, Jain R, Taylor MW, Douglas RG (2015). The nasal microbiota in health and disease: variation within and between subjects. Front. Microbiol..

[51] Liu CM (2015). *Staphylococcus aureus* and the ecology of the nasal microbiome. Sci. Adv..

[52] Weyrich LS (2014). Resident microbiota affect *Bordetella pertussis* infectious dose and host specificity. J. Infect. Dis..

[53] Zhang YY (2019). Commensal microbes affect host humoral immunity to *Bordetella pertussis* infection. Infect. Immun..

[54] Escapa IF (2018). New insights into human nostril microbiome from the expanded human oral microbiome database (eHOMD): a resource for the microbiome of the human aerodigestive tract. Msystems..

[55] Zmora N (2018). Personalized gut mucosal colonization resistance to empiric probiotics is associated with unique host and microbiome features. Cell.

[56] Maldonado-Gómez M (2016). Stable engraftment of *Bifidobacterium longum* ah1206 in the human gut depends on individualized features of the resident microbiome. Cell Host Microbe.

[57] Charles DD (2019). Development of a novel *ex vivo* nasal epithelial cell model supporting colonization with human nasal microbiota. Front. Cell. Infect. Microbiol..

[58] De Rudder C, Arroyo MC, Lebeer S, Van de Wiele T (2020). Dual and triple epithelial co-culture model systems with donor-derived microbiota and THP-1 macrophages to mimic host-microbe interactions in the human sinonasal cavities. Msphere.

[59] Audry M (2019). Airway mucus restricts *Neisseria meningitidis* away from nasopharyngeal epithelial cells and protects the mucosa from inflammation. Msphere.

[60] Stewart C, Torr EH, Mohd Jamili N, Bosquillon C, Sayers I (2012). Evaluation of differentiated human bronchial epithelial cell culture systems for asthma research. J. Allergy.

[61] Grainger CI, Greenwell LL, Lockley DJ, Martin GP, Forbes B (2006). Culture of Calu-3 cells at the air interface provides a representative model of the airway epithelial barrier. Pharm. Res..

[62] Kreft ME (2015). The characterization of the human cell line calu-3 under different culture conditions and its use as an optimized in vitro model to investigate bronchial epithelial function. Eur. J. Pharm. Sci..

[63] Schulz C (2017). THP-1-derived macrophages render lung epithelial cells hypo-responsive to *Legionella pneumophila* - a systems biology study. Sci. Rep..

[64] Singhal D, Foreman A, Bardy JJ, Wormald PJ (2011). *Staphylococcus aureus* biofilms: nemesis of endoscopic sinus surgery. Laryngoscope.

[65] Gevaert E (2017). Extracellular eosinophilic traps in association with *Staphylococcus aureus* at the site of epithelial barrier defects in patients with severe airway inflammation. J. Allergy Clin. Immunol..

[66] De Boeck I (2017). Comparing the healthy nose and nasopharynx microbiota reveals continuity as well as niche-specificity. Front. Microbiol..

[67] Lehtinen MJ (2018). Nasal microbiota clusters associate with inflammatory response, viral load, and symptom severity in experimental rhinovirus challenge. Sci. Rep..

[68] Köck R (2016). Persistence of nasal colonization with human pathogenic bacteria and associated antimicrobial resistance in the German general population. New Microbes New Infect..

[69] Ragupathy S (2014). Toll-like receptor 2 regulates the barrier function of human bronchial epithelial monolayers through atypical protein kinase c zeta, and an increase in expression of claudin-1. Tissue Barriers.

[70] Martens K, Hellings PW, Steelant B (2018). Calu-3 epithelial cells exhibit different immune and epithelial barrier responses from freshly isolated primary nasal epithelial cells in vitro. Clin. Transl. Allergy..

[71] Kohanski MA, Lane AP (2015). Sinonasal epithelial cell response to *Staphylococcus aureus* burden in chronic rhinosinusitis. JAMA Otolaryngol. Head Neck Surg..

[72] Rocha-Ramirez LM (2017). Probiotic *Lactobacillus* strains stimulate the inflammatory response and activate human macrophages. J. Immunol. Res..

[73] Matsubara VH (2017). Probiotic bacteria alter pattern-recognition receptor expression and cytokine profile in a human macrophage model challenged with *Candida albicans* and lipopolysaccharide. Front. Microbiol..

[74] Vareille-Delarbre M (2019). Immunomodulatory effects of *Lactobacillus plantarum* on inflammatory response induced by *Klebsiella pneumoniae*. Infect. Immun..

[75] Hatten KM (2015). Corticosteroid use does not alter nasal mucus glucose in chronic rhinosinusitis. Otolaryngol. Head Neck Surg..

[76] Clark JG, Kim KH, Basom RS, Gharib SA (2015). Plasticity of airway epithelial cell transcriptome in response to flagellin. PLoS ONE.

[77] Ramakrishnan VR, Gitomer S, Kofonow JM, Robertson CE, Frank DN (2017). Investigation of sinonasal microbiome spatial organization in chronic rhinosinusitis. Int. Forum Allergy Rhinol..

[78] Soyka MB (2012). Defective epithelial barrier in chronic rhinosinusitis: the regulation of tight junctions by IFN-gamma and IL-4. J. Allergy Clin. Immunol..

[79] Chauche C, McSorley HJ (2018). Basal instinct: persistence of barrier dysfunction. Immunity.

[80] Ramezanpour M, Moraitis S, Smith JLP, Wormald PJ, Vreugde S (2016). Th17 cytokines disrupt the airway mucosal barrier in chronic rhinosinusitis. Mediat. Inflamm..

[81] Saieg A, Brown KJ, Pena MT, Rose MC, Preciado D (2015). Proteomic analysis of pediatric sinonasal secretions shows increased muc5b mucin in CRS. Pediatr. Res..

[82] Ding GQ, Zheng CQ (2007). The expression of muc5ac and muc5b mucin genes in the mucosa of chronic rhinosinusitis and nasal polyposis. Am. J. Rhinol..

[83] Kim YJ (2014). Hypoxia-mediated mechanism of muc5ac production in human nasal epithelia and its implication in rhinosinusitis. PLoS ONE.

[84] Kiedrowski MR (2016). Development of an in vitro colonization model to investigate staphylococcus aureus interactions with airway epithelia. Cell. Microbiol..

[85] Cooksley C, Rosciol E, Wormald PJ, Vreugde S (2015). TLR response pathways in NuLi-1 cells and primary human nasal epithelial cells. Mol. Immunol..

[86] Rothen-Rutishauser B, Blank F, Muhlfeld C, Gehr P (2008). In vitro models of the human epithelial airway barrier to study the toxic potential of particulate matter. Expert Opin. Drug Metab. Toxicol..

[87] McCauley KB (2017). Efficient derivation of functional human airway epithelium from pluripotent stem cells via temporal regulation of Wnt signaling. Cell Stem Cell.

[88] Van Nevel S, Koetzsch S, Weilenmann H-U, Boon N, Hammes F (2013). Routine bacterial analysis with automated flow cytometry. J. Microbiol. Methods.

[89] Props R (2017). Absolute quantification of microbial taxon abundances. ISME J..

[90] Props R, Monsieurs P, Mysara M, Clement L, Boon N (2016). Measuring the biodiversity of microbial communities by flow cytometry. Methods Ecol. Evol..

[91] Vilchez-Vargas R (2013). Analysis of the microbial gene landscape and transcriptome for aromatic pollutants and alkane degradation using a novel internally calibrated microarray system. Environ. Microbiol..

[92] De Paepe K, Kerckhof FM, Verspreet J, Courtin CM, Van de Wiele T (2017). Interindividual differences determine the outcome of wheat bran colonization by the human gut microbiome. Environ. Microbiol..

[93] Kozich JJ, Westcott SL, Baxter NT, Highlander SK, Schloss PD (2013). Development of a dual-index sequencing strategy and curation pipeline for analyzing amplicon sequence data on the MiSeq Illumina sequencing platform. Appl. Environ. Microbiol.

[94] Schloss PD, Gevers D, Westcott SL (2011). Reducing the effects of PCR amplification and sequencing artifacts on 16S rRNA-based studies. PLoS ONE.

[95] Chao A (1984). Nonparametric estimation of the number of classes in a population. Scand. J. Stat..

