# Supplementary material for: Lacticaseibacillus casei AMBR2 modulates the epithelial barrier function and immune response in a donor-derived nasal microbiota manner
Source: Sci Rep. 2020 Oct 9;10:16939. doi: 10.1038/s41598-020-73857-9 (PMC7547715; doi:10.1038/s41598-020-73857-9)
Supplement: Supplementary file 1 — Supplementary Information. [file 41598_2020_73857_MOESM1_ESM.pdf]

Lacticaseibacillus casei AMBR2 modulates the epithelial barrier function and immune response in a donor-derived nasal microbiota manner

Charlotte De Rudder[1], Cristina Garcia-Tímermans[1], Ilke De Boeck[2], Sarah Lebeer[2], Tom Van de Wiele[1,*, ‡], Marta Calatayud Arroyo[1,3, ‡]

[1] Center for Microbial Ecology and Technology, Faculty of Bioscience Engineering, Ghent University

[2] Research Group of Environmental Ecology and Applied Microbiology,Department of Bioscience Engineering, University of Antwerp

[3] Group of Lactic Bacteria and Probiotics, Department of Biotechnology, Institute of Agrochemistry and Food Technology (IATA) - Spanish Research Council (CSIC), Valencia, Spain

[‡] These authors contributed equally to the work.

[*] To whom correspondence should be addressed:

email: Tom.VandeWiele@UGent.be

address: Center for Microbial Ecology and Technology, Coupure Links 653, Ghent University, B-9000 Ghent, Belgium

Supplementary Material

Figures

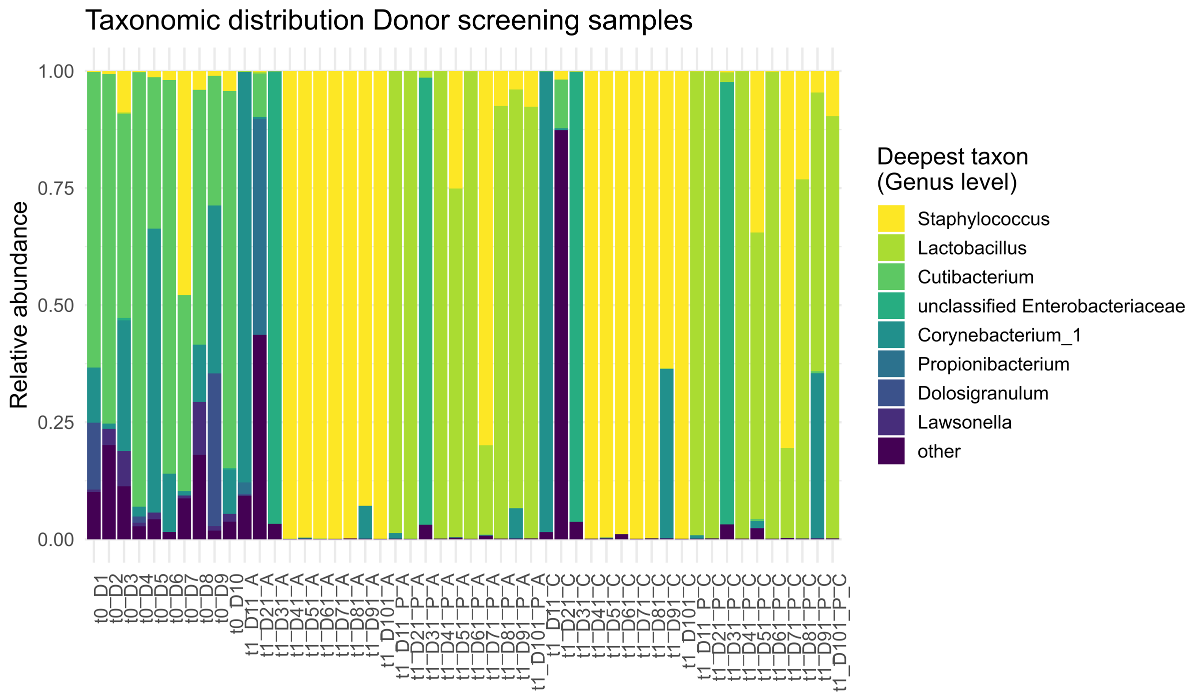

Figure 1: Relative abundance on genus level of the eight most abundant genera of nasal bacterial communities in swab samples (indicated with t0) and after 24 h of co-culture on a Calu-3 monolayer (indicated with t1) during the donor screening. Bacterial communities that were adhered to the cell layers are indicated with "C" and bacterial communities not adhered to the cell layers are indicated with "A". Numbers 1-10 indicate the different donors from which nasal microbiota were obtained.

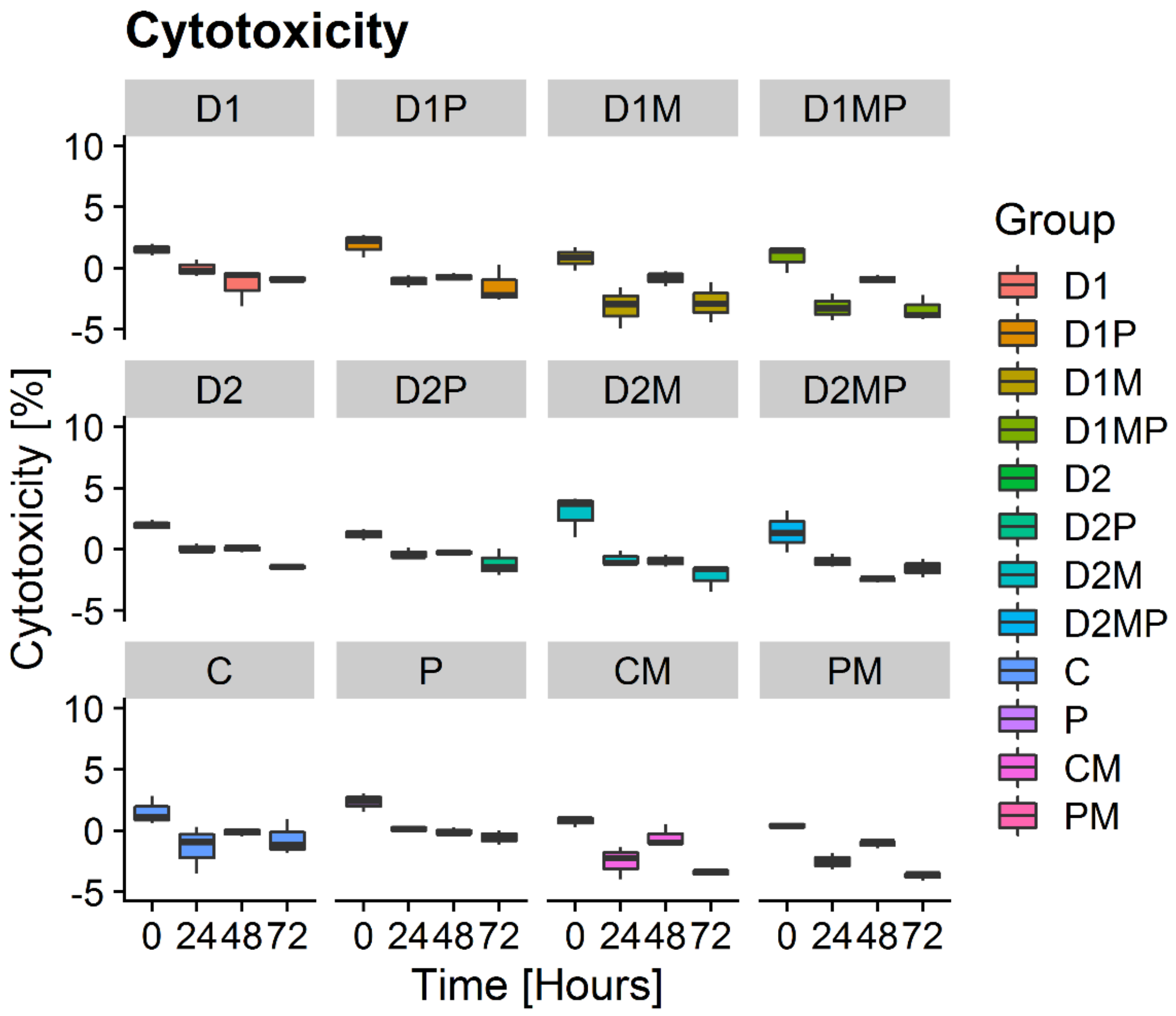

Figure 2: Cytotoxicity as determined by LDH release in dual and triple co-culture systems inoculated with nasal microbiota and/or *L. casei* AMBR2. LDH release is displayed as percentage cytotoxicity."D1" and "D2" indicate respectively inoculation with microbiota of donor 1 and donor 2, "M" indicates presence (triple) or absence (dual) of macrophage-like cells, "P" indicates presence of *L. casei* AMBR2, and "C" indicates the sterile control. Time is indicated in hours.

Tables

Table 1: S1: Characteristics of nasal swab donors incorporated in the screening assay. Sex (M: male/F: female), health status (Control: healthy control, CRS: chronic rhinosinusitis patient), smoker (Yes/No), age (years), BMI (body mass index, NA: information was not available – person with healthy BMI), ethnicity.

Donor	Sex	Health status	Smoker	Age	BMI	Ethnicity
D1	F	Control	No	28	18.5	Spanish / European
D2	M	Control	No	32	23.2	Belgian / European
D3	F	Control	No	26	17.4	Chinese / Asian
D4	F	Control	No	25	22.4	Belgian / European
D5	M	Control	No	25	19.8	Belgian / European
D6	F	Control	No	26	17.5	Belgian / European
D7	F	Control	No	24	23.1	Belgian / European
D8	F	Control	No	30	26.7	Mestizo
D9	F	Control	No	25	19.1	Belgian / European
D10	F	Control	No	29	NA	Chinese / Asian

Table 2: S2 - Part 1: Non-adhered bacteria. Number of events used to train and test the Random Forest classifier for each donor. Accuracy values and Kappa statistics are presented in %.

Donor	Events	Accuracy (%)	Kappa (%)
D1	30	97.8	95.6
D2	40	96.5	93.0
D3	2000	96.2	92.4
D4	50	93.5	86.9
D5	2000	98.7	97.4
D6	200	96.3	92.6
D7	2000	97.1	94.3
D8	2000	97.4	94.8
D9	2000	98.0	96.1
D10	2000	99.2	98.5

Table 2: S2 - Part 2: Adhered bacteria. Number of events used to train and test the Random Forest classifier for each donor. Accuracy values and Kappa statistics are presented in %.

Donor	Events	Accuracy (%)	Kappa (%)
D1	20	83.1	65.8
D2	20	81.0	62.1
D3	200	94.8	89.6
D4	80	84.8	69.6
D5	60	89.4	78.8
D6	20	84.0	67.9
D7	200	97.3	94.5
D8	90	82.0	63.9
D9	100	84.0	68.0
D10	200	92.6	85.2

Methods

Extracted DNA was first quantified using PicoGreen (QuantiFluor dsDNA kit, Promega). The amplicon PCRs included 3 or 10 μL of gDNA extract, 5X HOT BIOAmp® BlendMaster Mix – 12.5 mM $MgCl_2$ (Biofidal), 10X GC-rich Enhancer (Biofidal), with forward primer (341F) and reverse primer (785R) for the V3-V4 region of the 16S rRNA gene, and 20 mg.mL^{-1} bovine serum albumin, and had a final volume of 25 μL. 30 PCR cycles were done at 56 °C. The amplicon pools were purified using SPRIselect magnetic beads (Beckman Coulter) and eluted with 20 μL of nuclease-free H_2O. Purified PCR products were quantified using PicoGreen (Quantifluor dsDNA kit, Promega). Qualification of the purified amplicon PCR product was done on QIAxcel DNA screening cartridges (QIAGEN). The indexing PCR was done for 15 cycles using 2, 4, or 10 μL of the purified amplicon PCR product, 5X HOT BIOAmp® Blend Master Mix – 12.5 mM $MgCl_2$ (Biofidal) and 10X GC-rich Enhancer (Biofidal) in a final volume of 25 μL. Purification of the indexing PCR product was done using SPRIselect magnetic beads (Beckman Coulter) and the purified amplicon was eluted with 20 μL of nuclease-free H_2O. Purified PCR products were quantified using PicoGreen (Quantifluor dsDNA kit, Promega) and qualification of the purified amplicon PCR product was done on QIAxcel DNA screening cartridges (QIAGEN). This was followed by equimolar normalisation and pooling of the libraries. The sequencing pool was denatured using NaOH at 96 °C, and then diluted. The sequencing pool was loaded together with other external libraries (5 X phiX, non-indexed control library) and sequencing was done on an Illumina MiSeq in PE 2X300 bp using V3 chemistry (Illumina) by Biofidal (Vaulx-en-Velin, France – http://www.biofidal-lab.com).

The amplicon data were processed as described in [1] using mothur (v.1.39.5), largely based on the protocol by the Schloss lab [2, 3]. First, forward and reverse reads were assembled into contigs. Contigs with ambiguous base calls or non-sufficient overlap were removed. The remaining contigs were aligned to the mothur-formatted silva seed (release 123) alignment database, trimmed between positions 2 and 17012, to be compatible with the 341F (forward) and 785Rmod (reverse) primerset [4, 5]. Contigs not aligning with this region or containing homopolymer stretches of more than 12 bases, were removed. A preclustering step was then performed, clustering sequences with maximal 4 differences (1 % difference). A chimera check was performed using UCHIME [6] and chimeric sequences were removed. The sequences were then classified using the RDP 16S rRNA gene training set (version 16) by means of a naive Bayesian classifier (Wang's algorithm, Wang et al. [7]). Sequences classified as Eukaryota, chloroplasts and mitochondria were removed. Sequences not classifiable at (super)Kingdom level were removed as well. OTUs (operational taxonomic units) were clustered with an average linkage and at the 97 % sequence identity. Taxonomy assignment was

done using the RDP 16S rRNA gene training set (version 16) and silva database version 132. OTUs with only a single read were removed. Alpha diversity measures were calculated on samples that were sequenced sufficiently deep, as visually assed by levelling off of the rarefaction curves [8]. Collector curves were constructed for alpha diversity estimators and only stable estimators (collector curves independent of sequencing depth) were used for further analysis. Richness was estimated using the Chao1 index [9] and diversity was estimated the Inverse Simpson index. Samples from the dual and triple co-culture set-ups yielded insufficient biomass for sequencing, therefore only the swab samples (t= 0 h, inoculum) are displayed.

Bibliography

[1] K. De Paepe, F. M. Kerckhof, J. Verspreet, C. M. Courtin, and T. Van de Wiele. Inter-individual differences determine the outcome of wheat bran colonization by the human gut microbiome. *Environmental Microbiology*, 19(8):3251–3267, 2017. ISSN 1462-2912. doi: 10.1111/1462-2920.13819. URL <GotoISI>://WOS:000407790700025.

[2] J. J. Kozich, S. L. Westcott, N. T. Baxter, S. K. Highlander, and P. D. Schloss. Development of a dual-index sequencing strategy and curation pipeline for analyzing amplicon sequence data on the miseq illumina sequencing platform. *Applied and Environmental Microbiology*, 79(17): 5112–5120, 2013. ISSN 0099-2240. doi: 10.1128/Aem.01043-13. URL <GotoISI>://WOS:000322828100003.

[3] P. D. Schloss, D. Gevers, and S. L. Westcott. Reducing the effects of pcr amplification and sequencing artifacts on 16s rrna-based studies. *Plos One*, 6(12), 2011. ISSN 1932-6203. doi: ARTNe2731010.1371/journal.pone.0027310. URL <GotoISI>://WOS:000298369100004.

[4] A. Klindworth, E. Pruesse, T. Schweer, J. Peplies, C. Quast, M. Horn, and F. O. Gloeckner. Evaluation of general 16S ribosomal RNA gene PCR primers for classical and next-generation sequencing-based diversity studies. *Nucleic Acids Research*, 41(1), JAN 2013. ISSN 0305-1048. doi: {10.1093/nar/gks808}.

[5] C. Quast, E. Pruesse, P. Yilmaz, J. Gerken, T. Schweer, P. Yarza, J. Peplies, and F. Glöckner. The silva ribosomal rna gene database project: Improved data processing and web-based tools. *Nucleic acids research*, 41, 11 2013. doi: 10.1093/nar/gks1219.

[6] R. C. Edgar, B. J. Haas, J. C. Clemente, C. Quince, and R. Knight. UCHIME improves sensitivity and speed of chimera detection. *BIOINFORMATICS*, 27(16):2194–2200, AUG 15 2011. ISSN 1367-4803. doi: {10.1093/bioinformatics/btr381}.

[7] Q. Wang, G. M. Garrity, J. M. Tiedje, and J. R. Cole. Naive Bayesian classifier for rapid assignment of rRNA sequences into the new bacterial taxonomy. *APPLIED AND ENVIRONMENTAL MICROBIOLOGY*, 73(16):5261–5267, AUG 2007. ISSN 0099-2240. doi: {10.1128/AEM.00062-07}.

[8] J. Oksanen, F. G. Blanchet, M. Friendly, R. Kindt, P. Legendre, D. McGlinn, P. R. Minchin, R.B. O'Hara, G. L. Simpson, P. Solymos, M. H. M. Stevens, E. Szoecs, and H. Wagner. *vegan: Community Ecology Package*, 2018. URL https://CRAN.R-project.org/package=vegan. R package version 2.5-2.

[9] A. Chao. Nonparametric estimation of the number of classes in a population. *Scandinavian Journal of Statistics*, 11(4):265–270, 1984. ISSN 03036898, 14679469. URL http://www.jstor.org/stable/4615964.
